# *De Novo* Sequencing and High-Contiguity Genome Assembly of *Moniezia expansa* Reveals Its Specific Fatty Acid Metabolism and Reproductive Stem Cell Regulatory Network

**DOI:** 10.3389/fcimb.2021.693914

**Published:** 2021-07-06

**Authors:** Yi Liu, Zhengrong Wang, Wanlong Huang, Shuai Pang, Lingxiao Qian, Yanyan Zhang, Jimeng Meng, Mengfei Xu, Weiyi Wang, Yunfei Wang, Baoyan Lu, Yiyue Zhao, Jinwen Xian, Xinwen Bo, Bisong Yue

**Affiliations:** ^1^ Key Laboratory of Bio-Resources and Eco-Environment (Ministry of Education), College of Life Sciences, Sichuan University, Chengdu, China; ^2^ State Key Laboratory of Sheep Genetic Improvement and Healthy Production, Institute of Animal Husbandry and Veterinary, Xinjiang Academy of Agricultural and Reclamation Sciences, Shihezi, China; ^3^ NGS Research Center, Novogene Bioinformatics Institute, Beijing, China

**Keywords:** parasitology, worms, *Moniezia expansa*, genome, lipid, reproduction

## Abstract

*Moniezia expansa* (*M. expansa*) parasitizes the small intestine of sheep and causes inhibited growth and development or even death. Being globally distributed, it causes considerable economic losses to the animal husbandry industry. Here, using Illumina, PacBio and BioNano techniques, we obtain a high-quality genome assembly of *M. expansa*, which has a total length of 142 Mb, a scaffold N50 length of 7.27 Mb and 8,104 coding genes. *M. expansa* has a very high body fat content and a specific type of fatty acid metabolism. It cannot synthesize any lipids due to the loss of some key genes involved in fatty acid synthesis, and it may can metabolize most lipids *via* the relatively complete fatty acid β-oxidation pathway. The *M. expansa* genome encodes multiple lipid transporters and lipid binding proteins that enable the utilization of lipids in the host intestinal fluid. Although many of its systems are degraded (with the loss of homeobox genes), its reproductive system is well developed. PL10, AGO, Nanos and Pumilio compose a reproductive stem cell regulatory network. The results suggest that the high body lipid content of *M. expansa* provides an energy source supporting the high fecundity of this parasite. Our study provides insight into host interaction, adaptation, nutrient acquisition, strobilization, and reproduction in this parasite and this is also the first genome published in *Anoplocephalidae*.

## Introduction

*M. expansa* (Cyclophyllidea, Anoplocephalidae) is a parasitic flatworm with a cosmopolitan distribution and is mainly a parasite of Perissodactyla, Artiodactyla, and Primates (including humans) (Zhao et al., 2009). *M. expansa* mainly parasitizes the small intestine and harms host animals in three ways. First, the worms take in large amounts of nutrients from the host body and grow rapidly in the intestine, causing weight loss and weakness. Second, if a large number of eggs (such as cysticerci) are swallowed, the organisms can agglomerate during their development into adults, hindering the passage of chyme and causing intestinal blockage or even death. Third, the worms secrete large amounts of toxic substances during development that damage the host’s nervous system and clinically manifest as neurological symptoms in regions such as the head and back of the neck (Akrami et al., 2018).

*M. expansa* individuals can be up to 10 m long and are able to grow rapidly because the neck proglottids can continuously produce new proglottids to the rear. Individuals can grow up to 8~12 cm a day. Each proglottid carries a set of reproductive systems, and the sexual organs in gravid proglottids have degraded, with only the uterus fully developing and occupying the whole proglottid; thus, the individuals are said to be “egg-laying machines” (Gerald, 1986). Reproduction is one of the most important activities of an organism, and lipids play important physiological roles in this process. Stored lipids can be used as both a potential energy source and an active organ for the storage, metabolism and release of steroid hormones. The normal accumulation of total fat, triglycerides, phospholipids, and unsaturated fatty acids is critical to gonad development and embryonic and early larval growth. Triglycerides and phospholipids are the main lipid components of the gonads that provide energy for embryonic development (Sargent, 1995). In female humans, 22% body fat is necessary to maintain a menstrual cycle with ovulation, and a reduction in body fat below this threshold affects androgen conversion to estrogen under the catalysis of aromatase, leading to amenorrhea (Frisch, 1984). In the production of commercial pigs, body lipid loss is an important contributor to the low reproductive efficiency of many sows. Sows with “thin sow syndrome” become thin and neither show estrus nor successfully breed (Gatlin et al., 2002). During reproduction in corals, the lipid content greatly increases by up to approximately 85% to facilitate the breeding process (Leuzinger et al., 2003). *M. expansa* has a high lipid content, but it is unclear whether this high content is related to high fecundity in this species.

The lipid content of adults of *Schistosoma mansoni* (*S. mansoni*) is greater than 40%. In this species, the role of triglycerides during the life cycle is unclear, although it is known that they do not contribute to the formation of other lipids. Moreover, their use as an energy supply has not been determined. However, *S. mansoni* has lipases that can break down triglycerides, which may prevent excessive fatty acid concentrations in cells (Berriman et al., 2009). In *Echinococcus* spp., the fatty acid-binding protein (*FABP*) and antigen B gene families are the most expressed genes in the metacestode stage, suggesting that fatty acid intake is critical in these species (Tsai et al., 2013). Antigen B is the most abundant and immunogenic antigen produced in the larval stage (cytoplasm) of *Echinococcus granulosus* (*E. granulosus*). The structure and function of this lipoprotein have not been fully elucidated. *In vitro* studies have shown that antigen B apolipoprotein can bind fatty acids (Olson et al., 2012).

Enhancing our understanding of reproductive mechanisms in *M. expansa* is important for not only understanding the strong adaptability of parasites to their environments but also raising awareness of sheep health and reproductive diseases. However, little is known about the genes that regulate reproduction in *M. expansa*, representing a crucial gap in our knowledge of tapeworm biology. In this study, single molecule real-time sequencing (Pacific Biosciences; PacBio) and BioNano techniques were used to assemble the *M. expansa* genome for the first time. We examined genes and pathways possibly linked to the high body lipid content and high reproductive capacity of *M. expansa*. These data contribute to the growing global database for identifying parasites and parasite provenances. This study also provides insight into reproductive mechanisms and suggests some potential target molecules for effectively treating parasitic diseases.

## Materials and Methods

### Sample Information

Whole adult individuals from the intestines of freshly slaughtered sheep in the designated slaughterhouse for cattle and sheep in Shihezi City were sampled as the genome sequencing materials. The samples were placed in 37°C phosphate buffer brine (pH 7.4) in an insulated bucket and immediately transferred to the laboratory. The collected *Moniezia* individuals were classified as *Moniezia benedeni* (*M. benedeni*) or *M. expansa* based on the intersegmental glands following hematoxylin staining. In *M. expansa*, the intersegmental glands appear as a continuous row of circles distributed along the posterior edge of the internode. The transcriptome material datasets employed were from [https://dataview.ncbi.nlm.nih.gov/object/PRJNA542191] (SRA: PRJNA542191) (Liu et al., 2019).

### Genome Sequencing and Assembly

One PacBio library with an insert size of 20 kb and one BioNano library were constructed. The PacBio library was sequenced on a PacBio Sequel sequencer (Pacific Biosciences, Menlo Park, CA, USA). BioNano optical mapping was performed with Saphyr’s streamlined workflow (BioNano Genomics, San Diego, CA, USA). The genome size of *M. expansa* was estimated based on the k-mer spectrum. Using Jellyfish (v2.1.3) (Marçais & Kingsford, 2011), 17-mers were counted from short clean reads. With the long reads generated from the PacBio Sequel platform, contig assembly was carried out using the FALCON assembler (v1.2.4) (Pendleton et al., 2015). Then, the assembly from the PacBio data was polished by Quiver (smrtlink 5.0.1) (Chin et al., 2013). Heterozygous portions of the assembly were removed with Purge Haplotigs software (Roach et al., 2018). After filtering the data by molecule length and label density, high-quality labeled molecules were pairwise aligned, clustered and assembled into contigs with the BioNano Genomics assembly pipeline IrysSolve. Next, to create hybrid scaffolds, optical maps were aligned to PacBio assembled contigs and scaffolded with BioNano’s hybrid-scaffold tool. Consensus sequences of assemblies were then subjected to mapping of approximately 80X Illumina paired-end reads using BWA (v0.7.10-r789) (Li & Durbin, 2010) and were corrected by Pilon (v1.22) (Walker et al., 2014). Accuracy of the genome was assessed at the single-base level. Briefly, short reads generated by the Illumina platform were mapped to the *M. expansa* genome using BWA, and variant calling was performed with SAMtools (Li, 2011). The completeness of the genome assembly was assessed by two approaches as follows. Benchmarking Universal Single-Copy Orthologs (BUSCO) analysis (Simão et al., 2015) was performed by searching against the BUSCO metazoan_odb9 database (v3.0.2). Core Eukaryotic Genes Mapping Approach (CEGMA) analysis (Parra et al., 2007) was carried out based on a core gene set including 248 evolutionarily conserved genes from six eukaryotic model organisms.

### Genome Annotation

Two technologies, homologous comparison and *ab initio* prediction, were applied to annotate the repeated sequences within the *M. expansa* genome assembly. For homologous comparison, RepeatMasker (Bergman and Quesneville, 2007) and the associated RepeatProteinMask were employed for homolog comparison to align against the Repbase database and the TE protein database, respectively (Bao et al., 2015). For *ab initio* prediction, LTR_FINDER (Xu and Wang, 2007), Repeat Scout (Price et al., 2005) and Repeat Modeler (v2.1) (Smit and Hubley, 2015) were used for *de novo* construction of the candidate database of repetitive elements of the *M. expansa* genome. The repeated sequences were then annotated using RepeatMasker. Tandem repeat sequences were *ab initio* predicted using Tandem Repeats Finder software (v4.07b) (Benson, 1999).

Three approaches were applied to predict the protein-coding genes in the *M. expansa* genome, including homology-based prediction, *ab initio* prediction, and transcriptome-based prediction. For homologous annotation, protein sequences from 12 species were aligned to the *M. expansa* genome using TBLASTN with an e-value cutoff of 1e-5. (Species information is provided in **Supplementary Table 6**). The BLAST hits remaining from each query were then conjoined by Solar (v0.9.6) (Yu et al., 2006). Genewise (v2.4.1) (Birney et al., 2004) was used to predict the exact gene structure of the corresponding genomic region for each candidate gene extended upstream and downstream by 1000 bp. Homology predictions were denoted as “Homology-set”. For transcriptome-based prediction, RNA-seq data were assembled with Trinity (v2.0) (Grabherr et al., 2011), and the assembled sequences were then aligned to the *M. expansa* genome using Program to Assemble Spliced Alignments (PASA) (Haas et al., 2008). Gene models created by PASA were denoted as “PASA-T-set” (PASA Trinity set). In addition, RNA-seq reads were directly mapped to the genome using TopHat2 (v2.0.13) (Kim et al., 2013), and the mapped reads then were assembled into gene models (“Cufflinks-set”) by Cufflinks (v2.1.1) (Trapnell et al., 2012). For *ab initio* prediction, Augustus (v3.2.3) (Stanke and Morgenstern, 2005), GeneID (v1.4) (Guigó et al., 1992), GENESCAN (Burge and Karlin, 1997), GlimmerHMM (v3.0.4) (Majoros et al., 2004), and SNAP (v2013-11-29) (Korf, 2004) software programs were simultaneously employed for gene model prediction, in which Augustus, SNAP, and GlimmerHMM were trained by PASA-H-set gene models. After applying these three approaches, all the gene models were finally integrated by EvidenceModeler (v1.1.1) (Haas et al., 2008). The weights were set for each type of evidence as follows: PASA-T-set > Homology-set > Cufflinks-set > Augustus > GeneID = SNAP = GlimmerHMM = GENESCAN. To obtain information on the untranslated regions (UTRs) and alternative splicing variation, PASA was applied to update the gene models. Furthermore, predicted genes less than 50 amino acids in length, supported only by ab initio evidence or having expression values < 1 were filtered out.

Functional annotation of protein-coding genes in the *M. expansa* genome was performed based on homologous searches in the SwissProt, NR (from NCBI), InterPro and KEGG Pathway databases. The InterPro Scan tool (Jones et al., 2014) was applied in coordination with the InterPro database to predict protein function based on the conserved protein domains and functional sites. The SwissProt, NR and KEGG Pathway databases were mainly mapped by gene set to identify the best match for each gene.

In addition, the gene structures of noncoding RNAs in the *M. expansa* genome were predicted. Briefly, tRNAs were predicted using the t-RNAscan-SE tool (1.3.1) (Lowe & Eddy, 1997). rRNA sequences were predicted by searching against the invertebrate rRNA database using BLAST with an E-value of 1E-10. Small nuclear and nucleolar RNAs and miRNAs were annotated using the Infernal tool (v1.1rc4) (Nawrocki et al., 2009) based on the Rfam database.

### Phylogenetic Reconstruction and Divergence Estimation

Protein-coding sequences and protein sequences of 12 species were retrieved from the WormBase database (https://parasite.wormbase.org/) (**Supplementary Table 9**). For gene models with multiple alternative isoforms, only the longest transcript was selected to represent the gene. Subsequently, the all-against-all search algorithm with a cutoff of 1E-7 was implemented to identify orthologous gene relationships between *M. expansa* and other species, in which more than 30% coverage of the aligned regions in both orthologous genes was required. The alignments were clustered into gene families according to the OrthoMCL (Li et al., 2003) pipeline with the parameter “-inflation 1.5”.

The phylogenetic relationships between *M. expansa* and other species were reconstructed using the shared single-copy orthologous genes. The protein-coding sequences of the genes were aligned by the MUSCLE tool (Edgar, 2004) with default parameters. Sequences were then concatenated to one supergene sequence for each species and formed into a data matrix. Phylogenetic analysis was performed using the maximum-likelihood (ML) algorithm in RAxML (v8.0.19) (Stamatakis, 2006) with the GTR-GAMMA substitution model and with *C. elegans* and *Trichinella spiralis* as outgroups. The robustness of the maximum likelihood tree was assessed using the bootstrap method (100 pseudoreplicates). Furthermore, divergence times between *M. expansa* and other species were estimated using a Monte Carlo Markov chain algorithm implemented with the MCMCtree tool in the PAML package (v4.5). Three reference divergence time values (428.3~451.1 million years ago (MYA) for *C. elegans* and *T. spiralis*; 0.74~0.90 MYA for *T. saginata* and *T. asiatica*; and 492~1160 MYA for *C. elegans* and *S. mediterranea*) obtained from the TimeTree database (Kumar et al., 2017) were used to calibrate the divergence dates of other nodes on the phylogenetic tree.

### Expansion and Contraction of Gene Families

The evolutionary dynamics of gene families were analyzed with the CAFÉ tool (v4) (De Bie et al., 2006), which can identify gene families that have expanded or contracted using a stochastic birth and death model. The model can estimate the global parameter λ (based on the phylogenetic tree and the datasets of gene family clustering), which represents the birth and death rates of all gene families and identifies the significantly changed families. A *p*-value of 0.05 was taken as the threshold to identify significantly expanded or contracted gene families.

### Pathway Mapping

The metabolic and regulatory pathways of *M. expansa* were reconstructed on the basis of the KEGG Pathway database. The KEGG orthology identifier was used to link genes and pathways. The assignment of *M. expansa* genes to KEGG orthologs was performed with a modified bidirectional-best-BLAST-hits method.

### Analysis of Genes Involved in the Fatty Acid Synthesis Pathway

The *M. expansa* genome sequence assembly was searched by TBLASTN (E-value=10-5) with the amino acid sequences for all genes in the fatty acid synthesis pathway from a range of deuterostome genera as well as *S. japonicum*, *S. mansoni*, *Homo sapiens* (*H. sapiens*) and *Mus musculus* (*M. musculus*). The BLAST hits were then conjoined by Solar (v0.9.6) (Yu et al., 2006) and adjusted using phylogenetic information. The classification of deduced proteins and their integrity were verified using BLASTP against the NR database. Protein-domain information for other species was sourced from the Pfam database.

### Germ Cell Marker and Domain Analysis

Homologous sequences of other species downloaded from the NCBI database according to accession number were taken as the query sequences for identification of germ cell marker genes in the *M. expansa* genome. The candidate germ cell marker genes in the *M. expansa* genome were identified by BLASTP (E-value = 1e-5) software. In combination with domain (based on the Pfam database) and function (based on the NR database) annotation of the candidates, the final germ cell marker genes were identified. We determined whether the candidates had representative domains/motifs (Tsai et al., 2013; Milani et al., 2017) and known function annotations of germ cell marker genes. (The specific conserved domains/motif numbers are provided in **Supplementary Table 15**).

### *In Situ* Hybridization and Immunofluorescence

Paraffin sections of the scolex and neck together and of immature, mature and gravid proglottids were made. The four parts were all approximately 10 mm long. The scolex and neck samples included the scolex, neck and a few immature proglottids. The immature samples were 20-30 mm from the scolex. The mature samples were from approximately the middle of the strobila where both the male and female systems were mature, as confirmed by staining of the segments. The gravid samples were collected from the end of the strobila where the internal reproductive organs had degenerated and were full of eggs. The probe was synthesized in Guangzhou Exon Biotechnology Co., Ltd. For *in situ* hybridization, a rhodamine-labeled *PL10* probe was used. For immunofluorescence, endogenous peroxidase was inactivated, and the responsive samples were incubated with goat serum and goat anti-rabbit fluorescent secondary antibody. The secondary antibody is labeled with fluorescein isothiocyanate (FITC). PL10 is the identified evm.model.Contig51.208, and the primer sequence: 5 ‘GAAGCAAATCATCGGAAGC 3’ (F), 5 ‘CTCAAAACCCATGTCAAGC 3’ (R).

## Results and Discussion

### Genome Assembly and Annotation

A total of 30.02 Gb of PacBio (158X), 60.7 Gb of BioNano (319X) and 15.26 Gb paired-end reads derived from single adult worms were used for *M. expansa* genome assembly. The final assembly was approximately 142.23 Mb, with a contig N50 value of 3.39 Mb and a scaffold N50 value of 7.27 Mb (**Supplementary Tables 1** and **2**). The accuracy and completeness of the genome indicated high assembly integrity and sequencing uniformity. The mapping rate of reads from the small library was 95.79%, and the genome coverage was 99.20%. In addition, CEGMA analysis revealed that 222 of the 248 Core eukaryotic genes (CEGs) (89.52%) had been successfully assembled in the *M. expansa* genome. Similarity, BUSCO analysis revealed that 606 of the 978 metazoan BUSCOs were present in the genome assembly. This phenomenon of low BUSCO values exists throughout the platyhelminths, probably because of their sheer number of orthologous single-copy genes (**Table 1**). The GC content of *M. expansa* (38.82%) was similar to that of other members of Cestoda (35.2-43.8%, **Supplementary Table 3** and **Table 1**).

**Table 1:** The genomic features of 32 species published in platyhelminthes.

Species Name	Clade	N50	Genome Size	Coding genes	GC	CEGMA	BUSCO
*C.sinensis*	Trematoda	415,842	547M	13,634	44%	71.80%	70%
*E.caproni*	Trematoda	26,853	834M	18,607	—	52.42%	44.50%
*F.hepatica*	Trematoda	1,901,411	1203M	9,708	44.10%	47.98%	65.50%
*O.viverrini*	Trematoda	1,347,703	620M	16,356	43.80%	73.79%	72.70%
*S.curassoni*	Trematoda	13,843	344M	23,546	34.20%	63.71%	47.30%
*S.haematobium*	Trematoda	317,285	375M	11,140	34.20%	73.39%	65.30%
*S.japonicum*	Trematoda	174,764	402M	12,738	34.10%	69.76%	53.30%
*S.mansoni*	Trematoda	50,458,499	409M	10,144	35.50%	77.02%	74.70%
*S.margrebowiei*	Trematoda	35,167	367M	26,189	34.30%	74.60%	60%
*S.mattheei*	Trematoda	12,268	340M	22,997	34.10%	61.69%	44.60%
*S.rodhaini*	Trematoda	18,635	343M	24,089	34.40%	60.89%	42.50%
*T.regenti*	Trematoda	7,673	701M	22,185	—	52.82%	26.90%
*D.latum*	Cestoda	6,726	531M	19,966	—	49.60%	30%
*E.canadensis*	Cestoda	74,230	115M	11,432	—	92.74%	70.30%
*E.granulosus*	Cestoda	5,228,736	114M	10,245	41.90%	93.15%	69.60%
*E.multilocularis*	Cestoda	13,762,452	114M	10,663	42.20%	93.55%	70.20%
*H.taeniaeformis*	Cestoda	12,412	103M	11,649	43%	84.68%	60.10%
*H.diminuta*	Cestoda	49,752	165M	11,271	35.20%	88.71%	70.70%
*H.microstoma*	Cestoda	7,673,820	168M	10,139	36%	91.94%	73.10%
*H.nana*	Cestoda	19,213	162M	13,777	36.70%	87.10%	68.90%
M.expansa	Cestoda	7,274,224	142 M	8,104	38.82%	89.52%	62%
*M.corti*	Cestoda	65,816	117M	10,614	41.80%	91.53%	68.10%
*S.solidus*	Cestoda	31,505	539M	20,228	43%	76.61%	62.30%
*S.erinaceieuropaei*	Cestoda	4,624	1258M	39,557	—	29.44%	23%
*T.asiatica*	Cestoda	342,420	168M	13,322	43.10%	92.74%	69.50%
*T.multiceps*	Cestoda	44,815,576	240M	12,890	43.80%	87.90%	67.20%
*T.saginata*	Cestoda	585,232	169M	13,161	43.20%	93.15%	70.50%
*T.solium*	Cestoda	67,829	122M	12,481	42.90%	93.15%	69%
*G.salaris*	Monogenea	18,344	67M	15,436	33.90%	89.52%	66.30%
*P.xenopodis*	Monogenea	2,893	617M	37,906	—	39.52%	20.50%
*M.lignano*	Rhabditophora	244,885	764M	49,013	45.90%	99.19%	88.20%
*S.mediterranea*	Rhabditophora	40,740	901M	29,850	29.90%	91.13%	70.60%

According to WormBase database statistics, data for 15 species of Cestoda have been published, with the species concentrated in two orders (Pseudophyllidea and Cyclophyllidea), and the genome sizes of Pseudophyllidea (*D. latum*, 531 Mb; *Spirometra erinaceieuropaei*, 1258 Mb) are much larger than those of Cyclophyllidea (103~240 Mb). *M. expansa* belongs to Cyclophyllidea, in the family Anoplocephalidae, and the assembled genome size of *M. expansa* was 142 Mb, including 119 (84%) Mb unique contigs and 23.04 (16%) Mb repeats (**Supplementary Table 4**). The repeat sequences mainly included long interspersed nuclear elements (LINEs) (5.79%), long terminal repeats (LTRs) (4.16%), DNA transposons (0.69%) and short terminal repeats (SINEs) (0.11%) (**Supplementary Table 5**).

A total of 8,104 genes were obtained by homology-based, transcriptome-based and *ab initio*-based predictions (**Supplementary Table 6**). Of these genes, 97.70% (7,914) have homologues in public databases (**Supplementary Table 7**). Among the noncoding RNAs, 126 tRNAs, 12 rRNAs, 3 miRNAs and 178 snRNAs were identified (**Supplementary Table 8**).

### Comparative Genomic Analysis

**Figure 1:**
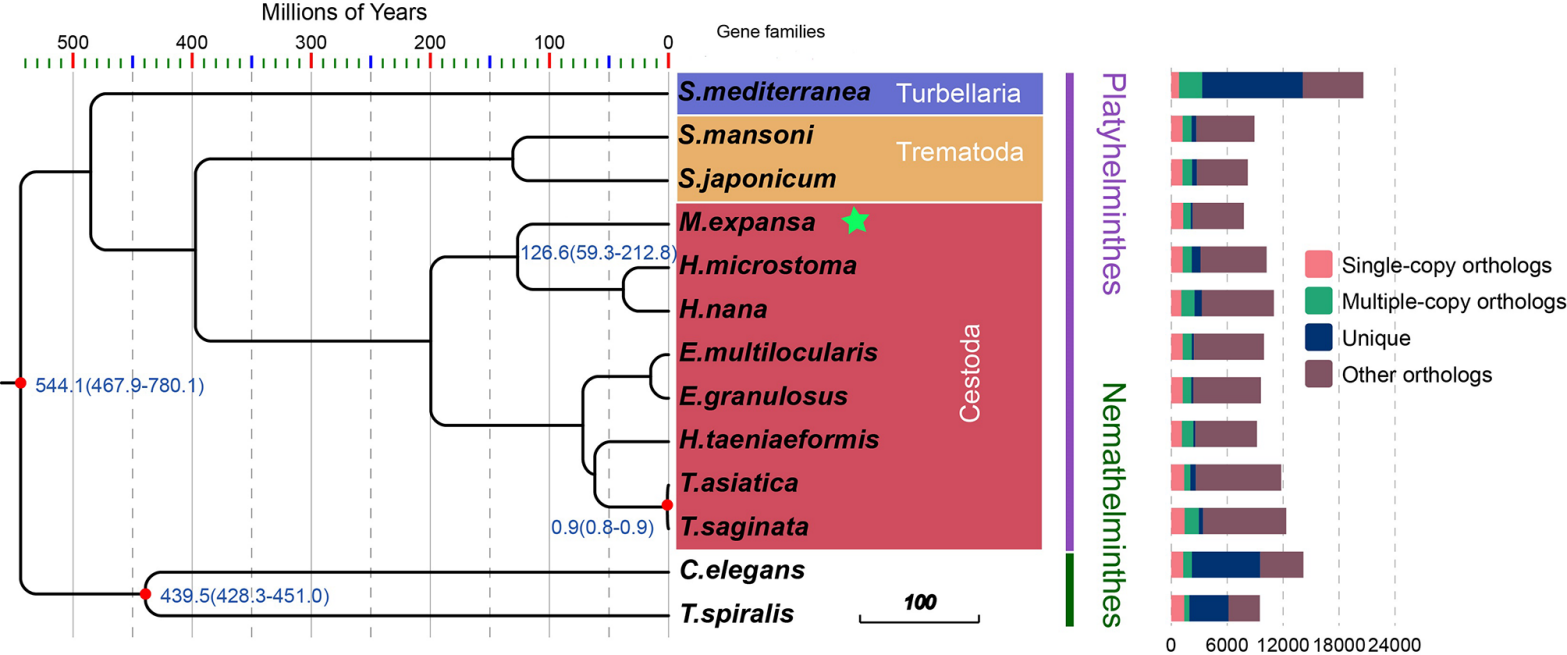
Phylogenetic tree and the distribution of genes in different species. Numbers in the branches are estimates of divergence time.

There were 21,832 gene families in 13 species and 386 single-copy gene families shared by all species (**Figure 1** and **Supplementary Table 9**). A total of 54 gene families were specific to *M. expansa* relative to other members of Cestoda (Blue legend of **Figure 1**). Interestingly, the KEGG enrichment results of these gene families were enriched in several pathways related to fatty acid transport and degradation, for instance, the pathways ABC transporters, Fatty acid degradation and Bile secretion, implying that its fatty acid metabolism is special (**Supplementary Figure 1**). Phylogenetic analysis suggested that *M. expansa*, *H. microstoma* and *H. nana* formed a branch with a divergence time of 126.6 million years, with a bootstrap support value of 100 (**Figure 1**). *M. expansa* and *H. microstoma* have similar life histories; their intermediate hosts are numerically dominant arthropod groups (armored mites and beetles), and the adult parasites live in the small intestines of mammals (Akrami et al., 2018). The positions of other species on the evolutionary tree were consistent with the results of other published articles (Wang et al., 2016).

### Specific Fatty Acid Metabolism

The key gene fatty acid synthase (*FASN*), fatty acid synthase subunit β (*FAS1*) and fatty acid synthase subunit α (*FAS2*) were not observed in *M. expansa* (**Supplementary Figure 2**). Although the key genes for fatty acid synthesis are lacking in in *M.* expansa, some genes related to fatty acid synthesis are present, for instance, acetyl-CoA carboxylase (*ACACA*), 3-oxoacyl-acyl-carrier protein reductase (*FabG*), enoyl-acyl-carrier protein reductase I (*FabI*), enoyl-acyl-carrier protein reductase III (*FabL*), 3-acyloxyacyl-acyl-carrier-protein synthase II (*FabF*) and acyl-carrier-protein S-malonyltransferase (*FabD*). *ACACA*, *FabD* and *FabF* also appear in *E. granulosus* (Zheng et al., 2013), whereas *S. japonicum* harbors only *FabF* (Schistosoma japonicum Genome Sequencing and Functional Analysis Consortium, 2009). Nevertheless *M.* expansa cannot synthesize fatty acids, it may can degrade fatty acids because of the relatively completeness of the fatty acid β**-**oxidation pathway (**Supplementary Figure 3**). A comparison of *Opisthorchis viverrini* (**Supplementary Figure 4**) has been added to the fatty acid degradation pathway. *O. viverrini* has relatively complete fatty acid degradation pathways for living in the bile, a fatty acid-rich environment as well as small intestine solution, while *Schistosoma mansoni* (**Supplementary Figure 5**) have only a few fatty acid degradation genes may for living in blood. The above reference data are all from KEGG (https://www.kegg.jp/).

In addition, *M.* expansa encodes a variety of lipid transporters and lipid binding proteins, including ATP-binding cassette (ABC) transporters, CD36 scavenger receptor long-chain fatty acid transporters, apolipoprotein-binding proteins, solute carrier families, low-density lipoprotein receptor proteins, phosphatidyl inositol transfer proteins, fatty acid binding proteins, and triglyceride transfer proteins, to utilize lipids in the host intestinal fluid (**Supplementary Table 11**). The expression of most lipid transporters and lipid binding proteins in mature adult and gravid proglottids was higher than that in the scolex, neck and immature proglottids (**Supplementary Table 11**). I-FABP (FABP2) is the most abundant fatty acid binding protein in *M.* expansa and is associated with the parasite’s ability to survive the environment of the small intestine (**Supplementary Figure 6**).

**Figure 2:**
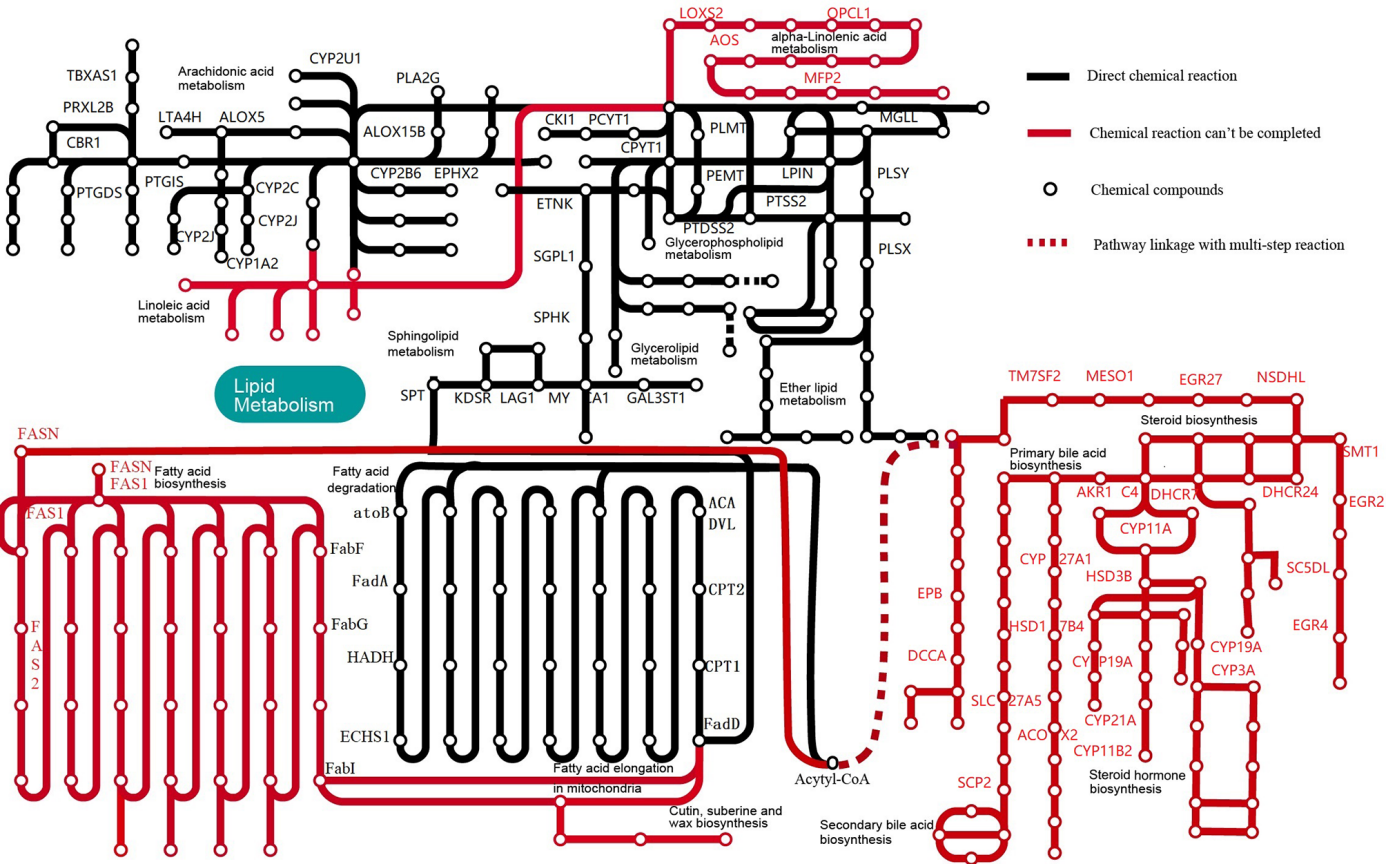
Lipid metabolic pathways in *M. expansa*. Black solid lines indicate direct chemical reactions, whereas red dashed arrows indicate that chemical reactions cannot be completed.

KEGG analysis showed that *M. expansa* can utilize carbohydrates, including glucose, through processes and pathways such as glycolysis/gluconeogenesis, the pentose phosphate pathway, phosphoinositide metabolism, and the citrate cycle (TCA cycle), which are all complete in *M. expansa*. The resulting NADH is used for ATP production by a complete mitochondrial electron transport system (the oxidative phosphorylation pathway). Although *M. expansa* cannot synthesize any lipids (including unsaturated fatty acids, steroids, and steroid hormones), it can metabolize most lipids, including clycerolipids, clycerophospholipids, ether lipids, sphingolipids and arachidonic acids (except linoleic acid and α-linolenic acid) (**Figure 2** and **Supplementary Table 10**). The fat content in *M. expansa* was detected by Soxhlet extraction and found to be very high (dry weight), and fifteen kinds of fatty acids were detected from 37 fatty acids according to national food safety standard GB5009.168-2016 (**Supplementary Table 12**). Although *M. expansa* cannot synthesize lipids, it can transport lipids from the small intestine of the host to its body through lipid transporters and lipid binding proteins to provide energy for mass reproduction.

In addition to examining pathways related to lipid metabolism, we examined pathways of nucleotide and amino acid metabolism. The metabolism of nucleotides (purine and pyrimidine) is indispensable. However, *M. expansa* cannot synthesize any of the following seven amino acids: valine, leucine, isoleucine, lysine, phenylalanine, tyrosine and tryptophan. The only amino acid that it can synthesize is arginine. Nevertheless, *M. expansa* can metabolize many amino acids, such as alanine, aspartic acid, glutamic acid, glycine, serine, threonine, cysteine, methionine, arginine, proline, histidine and tryptophan (but not lysine or phenylalanine) (**Supplementary Table 10**).

### Reproductive Stem Cell Regulatory Network

Vasa, Piwi and Nanos are thought to play conserved roles in reproductive stem cell maintenance and protection throughout the metazoan life cycle. Recent evidence in planarians supports these roles; furthermore, these genes have been shown to play roles in pluripotent stem cell maintenance and regeneration (Rebscher et al., 2012). In the present study, we use bioinformatics methods and experiments to explore the phylogeny and expression of these proteins in *M. expansa*. The numbers and specific gene numbers identified in each species are provided in **Table 2** and **Supplementary Table 14**.

**Table 2:** The numbers of reproductive stem cell marker genes identified in each species.

Species	Nanos		Argonaute				Royal family				RNase III		DEADbox	
	Nanos	Pumilio	AGO	Piwi	Group4	CHROMO		MBT	PWWP	Tudor	Dicer	Drosha	Vasa	PL10
*S.mediterranea*	0	2	0	8	1	8		5	9	4	1	2	1	2
*S.mansoni*	2	3	1	0	2	9		3	7	1	1	1	0	2
*S.japonicum*	2	5	1	0	2	9		4	8	1	1	1	0	1
*C. sinensis*	1	3	1	0	2	7		3	5	0	1	1	0	2
*F.hepatica*	1	3	1	0	1	5		5	6	1	1	1	0	0
*M.expansa*	1	3	1	0	3	8		2	7	1	2	1	0	1
*E.multilocularis*	2	3	1	0	3	7		2	5	2	3	1	0	2
*H.microstoma*	1	3	1	0	3	12		2	8	4	2	1	0	2
*E.granulosus*	2	3	1	0	2	7		2	5	2	2	1	0	1
*T.saginata*	2	3	1	0	2	6		2	7	1	3	2	0	1
*D.latum*	1	3	0	0	1	6		0	3	1	1	2	0	0
*C.elegans*	2	10	5	1	0	11		2	1	7	1	1	0	2
*D.melanogaster*	1	3	1	3	0	17		3	8	8	2	1	1	1
*B.rerio var*	3	3	5	2	0	40		15	31	11	2	1	1	2
*X.laevis*	6	6	7	4	0	45		11	33	8	2	2	2	2
*H.sapiens*	3	3	4	5	0	26		9	21	6	1	1	1	2
*O.aries*	3	3	4	4	0	25		8	18	5	1	1	1	1
*B.taurus*	2	3	3	4	0	22		8	22	3	1	1	1	1

We found only one member (evm.model.contig71.716) of the Nanos family in *M. expansa*. Phylogenetic trees constructed from the homologous sequences of 19 species did not show specific branches, indicating that Nanos members were conserved in the process of evolution. Three Pumilio family members (evm.model.contig489.35, evm.model.contig82.8 and evm.model.contig71.399) were identified in *M. expansa*. Both evm.model.contig82.8 and evm.model.contig71.399 had an obvious Puf domain (PUM-HD) (**Supplementary Figure 7**). The Pumilio phylogenetic tree showed three branches: one of all species, one of platyhelminths and one of species excluding platyhelminths. Evm.model.contig489.35 was found in all species, including higher vertebrates, arthropods, and platyhelminths. While evm.model.contig82.8 and evm.model.contig71.399 were identified as specialized sequences in platyhelminths (**Supplementary Figure 8**).

**Figure 3:**
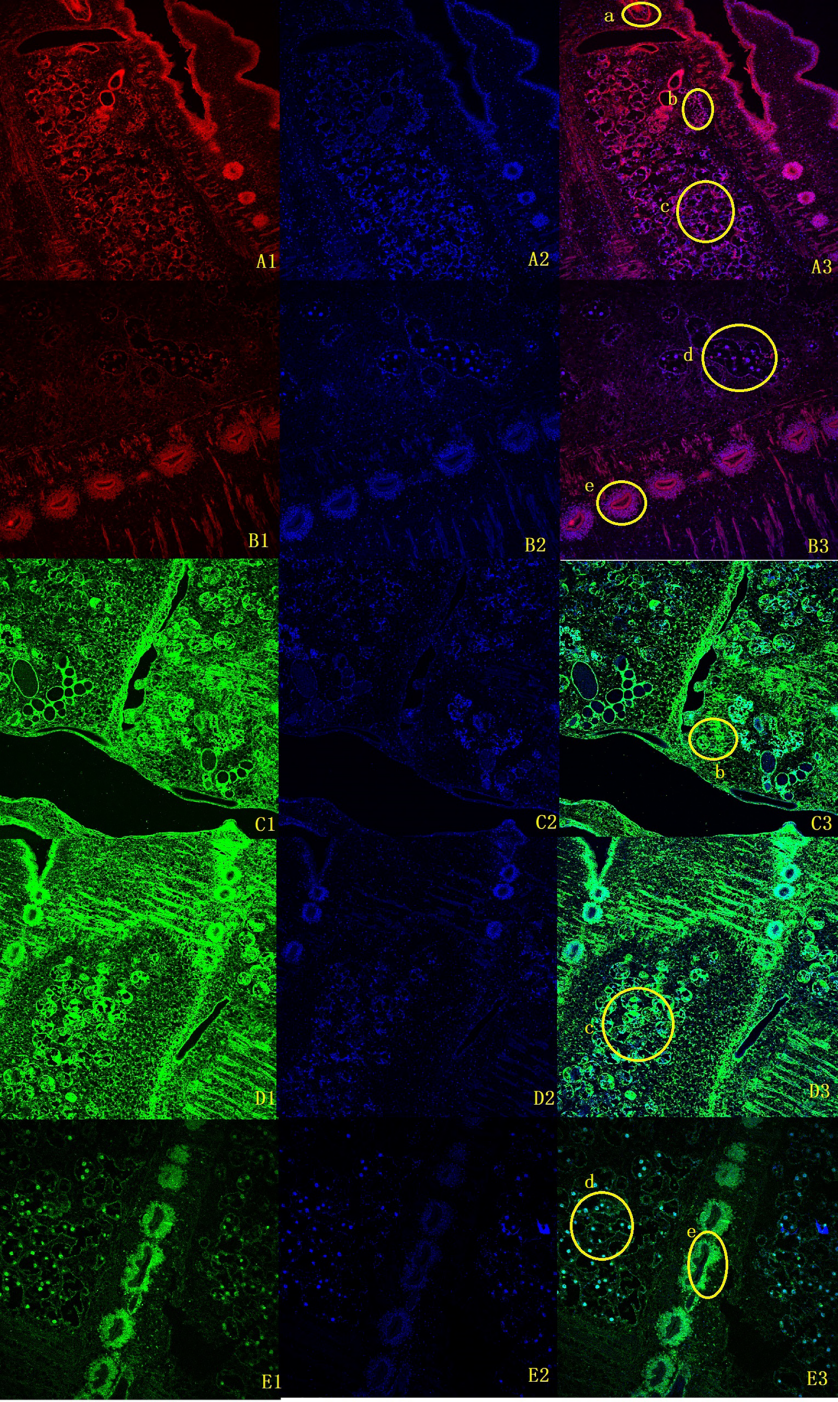
Fluorescence *in situ* hybridization and immunofluorescence of *M. expansa PL10* (10X). **(A1-B3)**, *in situ* hybridization results for *PL10*. **(A1, B1)** are images after rhodamine- labeled probe hybridization (red); **(A2, B2)** are images after DAPI stains the nucleus; and **(A3, B3)** are merged images. **(C1-E3)**, Immunofluorescence results for *PL10*. **(C1, D1, E1)** are images after FITC labeled goat anti-rabbit antibody binding (green); **(C2, D2, E2)** are images after DAPI staining; **(C3, D3, E3)** are merged images. **(a)**, Cirrus pouch; **(b)**, Vitelline gland; **(c)**, Testis; **(d)**, Eggs; **(e)**, Intersegmental gland.

To clarify the boundary between PL10 and Vasa, we searched for PL10 and Vasa copies in a representative range of multicellular animals, focusing on all published members of flatworms. Vasa members were obviously missing in *M. expansa* but were present in both Cestoda and Trematoda (**Supplementary Figure 9**). Evm.model.Contig51.208 was the only PL10 gene found in *M. expansa*. Fluorescence *in situ* hybridization was used to locate it in *M. expansa*. PL10 was found to be distributed in the vitelline gland, cirrus pouch, egg, and testis of mature proglottids. The immunofluorescence analysis showed that PL10 was distributed in the intersegmental gland, vitelline gland, egg, mature testis and gravid proglottids (**Figure 3**). The body of the worm has autofluorescence, so the standard for our judgment is that the part that clearly emits red and green light is determined to be positive, and it is judged to be negative if it is similar to the background. **Figure 3** showed that the expression in the intersegmental gland, epidermis and part of the reproductive organs was obvious red/green, which was confirmed as positive expression. In addition, we noticed that another branch of DEAD-box helicases, DDX5, was present in platyhelminths (**Supplementary Figure 9**). The existence of subfamilies other than Vasa may cause Vasa to become redundant in Cestoda and Trematoda.

The phylogeny showed that the AGO protein has split into AGO branches, Piwi branches, a *C. elegans* group 3 Argonaute branch and a new group 4 branch (**Supplementary Figure 10**), consistent with previous studies (Tsai et al., 2013). A difference between the present and previous phylogenies is that the new group 4 branch includes a copy of the free-living planarian *S. mediterranea* (mk4.000678.05), whereas previous results identify this clade as parasitic-flatworm specific. With the exception of *S. mediterranea*, Cestoda and Trematoda showed no members in Piwi branches. In addition, the group 4 Tudor proteins that interact with Piwi proteins (with at least two Tudor domains typically present) are found only in planarians, which is consistent with Piwi present in *S. mediterranea* (**Supplementary Figure 11**). Dicer and Drosha members are present in the *M. expansa* genome (**Supplementary Figure 12**).

We also identified some development-related signaling pathwangs: Wnt, Hedgehog, TGF-β, and Hippo signaling pathways (**Supplementary Table 15**). In each pathway, there were copies of all major components, including ligands and receptors, and the domains were recognizable and complete, indicating that the pathway was conserved.

### Loss of Homeobox Genes

Compared with those in other species, many homeobox genes have been lost in *M. expansa*. There are only 32 sequences in *M. expansa* (**Table 3**), whereas humans have 297 sequences, zebrafish have 344 sequences, *Drosophila* has 107 sequences, and *C. elegans* has 99 sequences (based on data from HomeoDB) (Zhong & Holland, 2011). Although *M. expansa* has lost some neurodevelopment-related homeobox genes, it has retained many others (*Lbx*, *Prrx*, *Pou4*, *Pou6*, *Rax*, and *Gsx*), a finding possibly related to its underdeveloped nervous system.

**Table 3:** Homeobox genes of *M. expansa*.

Classes	Genes in Families		Classes	Genes in Families	
ANTP	*Gbx*	evm.model.Contig71.147	PRD	*Hbn*	evm.model.Contig69.688.1
	*Gsx*	evm.model.Contig2.37		*Isx*	evm.model.Contig2.52
	*Hox2*	evm.model.Contig2.207		*Prrx*	evm.model.Contig71.48
	*Hox4*	evm.model.Contig61.128			evm.model.Contig75.347
	*Hox6-8*	evm.model.Contig62.24		*Rax*	evm.model.Contig96.3
		evm.model.Contig2.208		*Rhox*	evm.model.Contig71.1164
		evm.model.Contig71.148	LIM	*Lmx*	evm.model.Contig82.248
	*Meox*	evm.model.Contig2.140	POU	*Pou3*	evm.model.Contig70.15
	*Barx*	evm.model.Contig51.546		*Pou4*	evm.model.Contig82.3
	*Bsx*	evm.model.Contig82.133		*Pou6*	evm.model.Contig71.1317
	*Lbx*	evm.model.Contig82.336	TALE	*Irx*	evm.model.Contig52.180
		evm.model.Contig71.1090			evm.model.Contig51.91
	*Msx*	evm.model.Contig71.664			evm.model.Contig61.159
	*Nk2.1*	evm.model.Contig70.175		*Meis*	evm.model.Contig51.360
	*Tlx*	evm.model.Contig92.125	ZF	*Zfhx*	evm.model.Contig61.106
	*Vax*	evm.model.Contig52.232	Other	Unassigned	evm.model.Contig61.160

This table includes all the homeodomain containing gene models found in M. expansa genomes. Gene models containing a homeodomain that could not be confidently placed in any known Class are given the category “Other”.

## Discussion

### Loss of Key Genes Involved in Fatty Acid Synthesis

Loss of the *FASN* gene is observed throughout the platyhelminths, including free-living worms (Grohme et al., 2018). The fatty acid synthase encoded by *FASN* plays an important role in every step of the *de novo* synthesis of fatty acids. Its loss indicates the dependence of parasites on host lipids and is an adaptation to parasitic life. In addition to *FASN*, *FAS1* and *FAS2* are also key genes in fatty acid synthesis. The initial stage of fatty acid synthesis requires acetyl CoA and malonyl CoA to be covalently linked to a thiol group on the ACP reactive group to form acetyl ACP and malonyl-ACP, and *FAS1* encodes a key enzyme, fatty acid synthase β subunit, in this process. In the immediate fatty acid elongation process, acetyl ACP and malonyl-ACP undergo continuous condensation, reduction, dehydration and reduction of the two-carbon unit, which requires the fatty acid synthase β subunit and the fatty acid synthase α subunit. Therefore, *M.* expansa cannot synthesize fatty acids without *FAS1* and *FAS2*.

Except for *FabL*, the other 5 enzymes (*ACACA*, *FabG*, *FabI*, *FabF* and *FabD*) in *M.* expansa are all necessary for FASII pathway of fatty acid synthesis. The FASII pathway of the apicoplast begins with the import of substrates from the cytoplasm, and through a series of reactions involving nine separate enzymes and the acyl carrier protein (*ACP*), results in the production of saturated fatty acids eight or more carbons in length (Shears et al., 2015). *M. expansa* belongs to the platyhelminthes and lacks the unique apicoplast structure of the *Apicomplexa*. Even the *Theileria* spp with apicoplast, are missing from some apicoplast proteins identified in *Plasmodium* spp., such as the enzymes for FASII pathway of fatty acid synthesis and heme and other housekeeping proteins such as SufC involved in plastidic Fe-S cluster assembly system (Sato, 2011). In addition, the enzymes found in *M. expansa* only occupies part of the FASII pathway, and some enzymes of the elongation phase of FASII extend the growing fatty acid by two carbons per cycle were absent in *M. expansa*, for instance, the 3-oxoacyl-[acyl-carrier-protein] synthase III (*FabH*), 3-oxoacyl-[acyl-carrier-protein] synthase I (*FabB*), 3-hydroxyacyl-[acyl-carrier-protein] dehydratase (*FabZ*). The **Supplementary Figure 2** showed the specific fatty acid synthesis pathways of *M. expansa*, including the FASII pathway that exists in the *Apicomplexa* parasites. So we found that *M. expansa* still does not have the ability to synthesize fatty acid *de novo*. Therefore, *M.* expansa has lost the ability to synthesize fatty acids to adapt to its living environment but has retained some genes related to fatty acid synthesis, presumably to modify the fatty acid chain.

### The Relatively Complete Fatty Acid β-Oxidation Pathway

*O. viverrini* (Young et al., 2014), which parasitizes bile, a lipoprotein-rich environment, can use lipids to provide energy, but other trematodes, such as *S. japonicum* (Schistosoma japonicum Genome Sequencing and Functional Analysis Consortium, 2009), *S. mansoni* (Berriman et al., 2009) and *S. haematobium* (Young et al., 2012), cannot complete the β-oxidation of fatty acids. *S. japonicum* has only enol-CoA hydratase (*echA*) and 3-hydroxyacyl-CoA dehydrogenase (*HADH*), and both *S. mansoni* and *S. haematobium* contain only long-chain acyl-CoA synthetase (*ACSL*) and acetyl-CoA-C-acetyl transferase (*atoB*). These organisms are all parasitic in the host’s blood and directly absorb small-molecule nutrients in veins from the intestine of mammalian hosts, so they do not have the ability to degrade long-chain fatty acids. The presence of the relatively complete fatty acid β-oxidation pathway in the *M.* expansa genome may contribute to the parasite’s use of the long-chain fatty acids in its host intestinal. The intestinal solution is rich in high-, medium-, low- and very low-density lipoproteins composed of different proportions of phospholipids, cholesterol and triglycerides.

### *M. expansa* Encodes Multiple Lipid Transporters and Lipid Binding Proteins

The main function of fatty acid binding proteins is to transport fatty acids, especially polyunsaturated fatty acids. The FABP family is divided into four broad categories. The first category consists of vitamin A derivative-specific binding proteins, including intracellular retinoid binding proteins (*CRABPI* and *CRABPII*) and intracellular retinol binding proteins (*CRBPI*, *CRBPI*I, *CRBPIII*, and *CRBPI*V). The second type of FABP generally binds to larger ligands, such as bile acids, heme, and eicosanoids, including (ileum) I-LBP, (liver) L-FABP, and (liver basic) Lb-FABP. The third type of FABP has only one member (intestinal) I-FABP. The fourth type of FABP includes (heart) H-FABP, (adipocyte) AFABP, (epidermal) E-FABP, (myelin) M-FABP, (testis) T-FABP, and (brain) B-FABP (Haunerland & Spener, 2004). Many of the FABP families identified in the genome of *M.* expansa are I-FABP. In addition, previous studies have indicated that the processes of reproductive organ maturation and oviposition need abundant lipids (Leuzinger et al., 2003). The high expression of lipid transporters and lipid binding proteins in *M. expansa* may help meet the lipid requirements of these processes.

### *Nanos* and *Pumilio* Genes Combine to Regulate Translation

In many species, Nanos combines with Pumilio to regulate translation. Pumilio targets mRNA through its PUM-HD, binding to the 3’ untranslated region (UTR) of the mRNA (Ewen-Campen et al., 2010). PUM-HD is evolutionarily conserved across species and is usually composed of eight tandem repeats, each consisting of 35-39 bases (Wang et al., 2002). *In situ* hybridization experiments showed that Pumilio is mainly distributed in the ovary and yolk gland of female *S. japonicum*. Upon silencing of Pumilio, obvious yolk gland atrophy was seen, and the number of eggs was reduced (Xia et al., 2020).

### PL10 Is Necessary for Germ Cell Formation

DEAD-box helicases are named after the Asp (D)-Glu (E)-Ala (A)-ASP (D) motif in their amino acid chain (Cordin et al., 2006). DEAD-box helicases includes two particularly important stem cell markers, Vasa and PL10. The Vasa and PL10 subfamilies are thought to be closely related, and phylogenetic evidence suggests that Vasa members are derived from the existing related ancestor of the PL10 family (Skinner et al., 2012). Given that free-living flatworms have Vasa copies, previous authors have speculated that the loss of Vasa was a unique loss that occurred in the common ancestor of Cestoda and Trematoda (Tsai et al., 2013). However, the presence of Vasa in flatworms does not seem necessary for their gonadal formation or stem cell proliferation (De Mulder et al., 2009). PL10 has the same DEAD and helicase C domain combination as Vasa. The present of PL10 in Cestoda and Trematoda predict that in Cestoda and Trematoda, Vasa is not essential for germ cells and that PL10 might have a role equally important as that of Vasa in most metazoan animals, which will be the subject of future investigations.

### The Absence of the Piwi Family

The Argonaute family of proteins is characterized by the existence of two domains: the Piwi domain and the PAZ domain. The Piwi domain can promote ribonuclease folding, creating pockets in which small RNA can be stored. The PAZ domain creates a hydrophobic pocket to bind to the 3’ end of the RNA (Batista & Marques, 2011). AGO binds siRNA and miRNA, and these RNAs require processing into mature small RNAs by the ribonuclease III enzyme Dicer and may require initial processing by the related enzyme Drosha (Wei et al., 2012). In contrast, Piwi only combines piRNA and rasiRNA, and one of the key characteristics of Piwi and piRNA is that throughout the animal kingdom, they have been shown to be almost exclusively associated with the germline (Houwing et al., 2007). Although Piwi was absent in Cestoda and Trematoda, the royal family proteins, which play a role in piRNA biosynthesis, were almost all present. The royal family’s retention suggests that the piRNA approach may still be functional in *M. expansa.*

### Development-Related Signaling Pathways

The Wnt signaling pathway can accurately guide regeneration in planarians, regulate the formation of the front and rear (AP) axis, and maintain gene gradient expression along the AP axis in muscle cells and stem cells (Lin & Pearson, 2014). Generally, compared to those without active cell differentiation, cells and tissues with active cell differentiation, such as osteoblasts, kidney, bone marrow, and fetal liver hematopoietic cells, often contain higher levels of TGF-β signals, and TGF-β signals can be detected in almost all tumor cells. The Hedgehog signaling pathway is one of the major regulators of embryonic development and tissue homeostasis in multicellular organisms (Zhu et al., 2020). The Hippo pathway is an evolutionarily conserved signaling cascade that controls organ size during development by regulating cell proliferation and apoptosis and stem cell self-renewal ability (Yin et al., 2020). Germline stem cells may transmit signals to the nucleus through receptors on the cell membrane *via* these four signaling pathways. On the other hand, the DEAD-box helicases and Argonaute families protect DNA from transposons during transcription, and Nanos and Pumilio combine to regulate the next translation process.

### Loss of Homeobox Genes-Degradation of Other Systems Except for the Reproductive System

The loss of homeobox genes is inseparable from parasitic life; such genes include *CDX* and *Pou5*, which are associated with intestinal mucosal and gastric mucosal development; *Hox5* and *Pitx*, which play roles in lung morphogenesis; *Hox9-13*, *Pax2/8* and *Hnf1*, which are expressed during kidney development; the main control genes *Pdx* and *Isl*, which are expressed in the pancreas; *Hhex* transcriptional regulators, which are involved in vascular development; *Hox3*, *Six1/2*, *Pax6*, *Prox* and *Vsx*, which are related to eye development; *Emx*, *Prop* and *Lhx6*, which are associated with taste phenotypic expression; *Mkx*, *Tshz1* and *Pax3/7*, which regulate muscle development to determine muscle fiber type; *Otp*, *Pax4* and *Pou1*, which are associated with the secretion of various hormones; *Six4/5* and *Lhx2/9*, which control the differentiation of stem cells into the olfactory cortex; *Tshz1*, *Shox* and *Uncx*, which are important for cartilage development; *Lhx6/8* and *Zeb*, which direct the differentiation of undifferentiated odontogenic mesenchymal cells into preodontoblast cells; *Otx*, which is associated with photoreceptor neurons; and *Evx*, *Dlx* and *Uncx* genes, which are associated with neuronal differentiation. The remaining *M. expansa* homeobox genes also play roles in a variety of activities. *Meox* is expressed in the mesoderm, epithelial segment and cutaneous muscle segment during development; *Gbx* promotes the reprogramming of mouse mesoderm stem cells to a state similar to that of mouse embryonic stem cells; *ISX* maintains immunity and tolerance; *Rhox* plays important roles in the occurrence, development and differentiation of the reproductive system; and *Barx*2 plays key roles in postnatal myocyte formation, including muscle maintenance during senescence. The attached table (**Supplementary Table 14**) provides functional references corresponding to the abovementioned homeobox genes.

## Conclusions

We applied Illumina, PacBio and BioNano technologies to conduct genome assembly of *M. expansa*, yielding a final genome with a total length of 142 Mb, a contig N50 value of 3.39 Mb and a scaffold N50 value of 7.27 Mb. The genome contains 8,104 protein-coding genes, and 16% of the genome is composed of repeat sequences. Among the studied taxa, *H. microstoma* had the closest evolutionary relationship with *M. expansa*, with the two lineages differentiating 66.4 MYA. Although the body fat content of this species is high, it cannot synthesize any lipids because of its lack of *FASN*, *FAS1* and *FAS2* genes, which are involved in fatty acid synthesis. *M.* expansa has the relatively complete fatty acid β**-**oxidation pathway and can metabolize most lipids through lipid transporters and lipid binding proteins to utilize lipids present in the host intestinal fluid. In adapting to parasitic life, *M.* expansa has undergone degradation of many of its systems, although not its reproductive system. PL10, AGO, Nanos and Pumilio, which play conserved roles in reproductive stem cell maintenance and protection, weave their powerful reproductive regulatory networks together with the Wnt, Hedgehog, TGF-β, and Hippo signaling pathways. The *M.* expansa genome sequences provided in this study enhance our understanding of the Anoplocephalidae.

## Data Availability Statement 

The whole genome shotgun project of *M. expansa* has been deposited at NCBI under BioProject PRJNA668441. The raw sequencing reads of DNA are available at SRA (SRR12858246-SRR12858252). The genome assembly data have been deposited at GenBank under accession no. JADFDV000000000.

## Author Contributions

XB conceived and designed the study. YL wrote the paper. ZW and LQ performed the experiment. WH and SP conducted the bioinformatics analysis. YZhang, JM, and MX participated in sampling and sample quality testing. WW, YW and BL interpreted the data. YZhao and JX completed the database query. BY revised the manuscript. All authors contributed to the article and approved the submitted version.

## Funding

Project support was provided by the National Key Basic Research Program (973 Program) of China (Grant no. 2015CB150300), the National Natural Science Foundation of China (Nos. 31860701 and 31360608), and the International Scientific and Technological Cooperation Projects of Xinjiang Production and Construction Corps (2017BC003).

## Conflict of Interest

Authors WH and SP were employed by the company Novogene Bioinformatics institute.

The remaining authors declare that the research was conducted in the absence of any commercial or financial relationships that could be construed as a potential conflict of interest.
