# Supplementary material for: De Novo Sequencing and High-Contiguity Genome Assembly of Moniezia expansa Reveals Its Specific Fatty Acid Metabolism and Reproductive Stem Cell Regulatory Network
Source: Front Cell Infect Microbiol. 2021 Jul 6;11:693914. doi: 10.3389/fcimb.2021.693914 (PMC8291045; doi:10.3389/fcimb.2021.693914)
Supplement: Supplementary file 1 [file DataSheet_1.zip › Supplementary material.docx]

## De novo sequencing and high-contiguity genome assembly of *Moniezia expansa* reveals its specific fatty acid metabolism and reproductive stem cell regulatory network

Yi Liu^1†^, Zhengrong Wang^2†^, Wanlong Huang^3†^, Shuai Pang^3^, Lingxiao Qian ^2^, Yanyan Zhang^2^, Jimeng Meng^2^, Mengfei Xu^2^, Weiyi Wang^2^, Yunfei Wang^2^, Baoyan Lu^2^, Yiyue Zhao^2^, Jinwen Xian^2^, Xinwen Bo^2^*, Bisong Yue^1^*

Supplementary Table 1. Statistics of *M.expansa* genome sequencing data

| Pair-end libraries | Insert size | Total data ( G) | Read length (bp) | Sequence coverage (X) |
| --- | --- | --- | --- | --- |
| Pacbio reads | 20 kb | 30.02 | -- | 158 |
| Bionano | -- | 60.7 | -- | 319.47 |
| Illumina reads | 350bp | 15.26 | 150 | 80.32 |

Supplementary Table 2. Overview of *M.expansa* genome assembly results

| Sample ID | Length | | Number | |
| --- | --- | --- | --- | --- |
|  | Contig^a^(bp) | Scaffold(bp) | Contig^a^ | Scaffold |
| Total^b^ | 136,538,930 | 142,226,486 | 579 | 552 |
| Max | 11,739,020 | 18,926,191 | - | - |
| Number>=2000 | - | - | 463 | 437 |
| N50 | 3,386,365 | 7,274,224 | 14 | 7 |
| N60 | 2,613,211 | 4,561,768 | 18 | 10 |
| N70 | 2,344,323 | 3,810,382 | 24 | 13 |
| N80 | 1,438,009 | 2,877,197 | 32 | 17 |
| N90 | 428,823 | 1,053,056 | 48 | 26 |

^a^ Contig after scaffolding

^b^ Assembly results select >100bp scaffolds

Supplementary Table 3. *M.expansa* genome base content statistics

| Base | Number (bp) | % of genome |
| --- | --- | --- |
| A | 41,755,414 | 29.36 |
| T | 41,775,593 | 29.37 |
| C | 26,506,862 | 18.64 |
| G | 26,501,367 | 18.63 |
| N | 5,687 | 4.00 |
| Total (bp) | 142,226,792 | 100 |
| GC | 53,008,229 | 38.82 |

* GC content of the genome without N

Supplementary Table 4. Repeated sequence statistics for different databases

| Type | Repeat Size(bp) | % of genome |
| --- | --- | --- |
| TRF | 2,848,887 | 2.00 |
| RepeatMasker | 22,718,472 | 15.97 |
| RepeatProteinMask | 2,073,210 | 1.46 |
| Total | 23,040,911 | 16.20 |

Note: Total is the non-redundant results after of removing the overlapping parts between the above various methods.

Supplementary Table 5. Repeated sequence classification statistics

| Denovo+Repbase | | | TE Proteins |  | Combined TEs | |
| --- | --- | --- | --- | --- | --- | --- |
|  | Length  (bp) | %in  Genome | Length  (bp) | % in  Genome | Length  (bp) | % in  Genome |
| DNA | 968,064 | 0.68 | 36,852 | 0.03 | 987,345 | 0.69 |
| LINE | 7,613,746 | 5.35 | 2,021,588 | 1.42 | 8,237,836 | 5.79 |
| LTR | 5,900,828 | 4.15 | 14,944 | 0.01 | 5,913,284 | 4.16 |
| SINE | 150,264 | 0.11 | 0 | 0 | 150,264 | 0.11 |
| Simple repeat | 1,320,892 | 0.93 | 0 | 0 | 1,320,892 | 0.93 |
| Unknown | 9,938,191 | 6.99 | 0 | 0 | 9,938,191 | 6.99 |
| Total | 22,718,472 | 15.97 | 2,073,210 | 1.46 | 22,764,383 | 16.01 |

Note: Denovo+Repbase is a transposon element derived from the RepBase nucleic acid library predicted by the RepeatModeler, RepeatScout and LTR_FINDER software in combination with the RepBase nucleic acid library using Uclust's software in accordance with the 80-80-80 principle and using the RepeatMasker software to annotate the genome; TE proteins is a transposon component obtained by annotating the genome with the RepeatProteinMask software based on The RepBase Protein Library; Combined TEs is the result of integrating the above two methods and de-duplicating. Unknown indicates that the repeat sequence cannot be classified by RepeatMasker.

Supplementary Table 6. *M.expansa* gene structure prediction through three methods

| Gene set | | Number | Average gene length (bp) | Average CDS length  (bp) | Average exons  per gene | Average exon length  (bp) | Average intron  length (bp) |
| --- | --- | --- | --- | --- | --- | --- | --- |
| Augustus | | 6,944 | 6,621.08 | 1,796.79 | 6.79 | 264.58 | 833.04 |
| GlimmerHMM | | 19,504 | 6,005.92 | 826.18 | 3.80 | 217.52 | 1,851.10 |
| *De novo* | SNAP | 15,788 | 4,614.44 | 1,003.69 | 5.05 | 198.78 | 891.71 |
|  | Geneid | 4,660 | 22,044.88 | 1,046.54 | 7.13 | 146.69 | 3,422.96 |
|  | Genscan | 9,039 | 10,309.40 | 1,697.12 | 6.75 | 251.47 | 1,498.11 |
|  | Emu | 8,387 | 5,080.50 | 1,528.18 | 5.84 | 261.79 | 734.33 |
|  | Egr | 7,852 | 4,937.63 | 1,512.00 | 5.70 | 265.14 | 728.45 |
|  | Hmi | 8,442 | 4,947.38 | 1,505.39 | 5.75 | 261.86 | 724.82 |
|  | Tas | 8,151 | 4,582.61 | 1,468.03 | 5.37 | 273.13 | 711.94 |
|  | Tsa | 6,804 | 6,841.94 | 1,776.49 | 6.69 | 265.60 | 890.46 |
| Homolog | Tso | 9,399 | 3,887.56 | 1,306.53 | 4.89 | 267.28 | 663.81 |
|  | Mli | 4,095 | 2,916.46 | 989.92 | 3.84 | 257.95 | 678.93 |
|  | Sma | 6,142 | 2,822.14 | 1,011.99 | 3.80 | 266.31 | 646.47 |
|  | Oar | 3,730 | 2,728.44 | 972.99 | 3.72 | 261.66 | 645.74 |
|  | Hsa | 3,763 | 2,732.06 | 987.39 | 3.78 | 261.25 | 627.70 |
|  | Mmu | 3,795 | 2,807.70 | 992.09 | 3.83 | 259.10 | 641.79 |
|  | Clu | 3,684 | 2,787.93 | 987.24 | 3.78 | 260.96 | 647.01 |
| RNAseq | PASA | 30,278 | 4,495.39 | 1,300.16 | 6.26 | 207.76 | 607.68 |
| Cufflinks | | 16,399 | 6,317.05 | 1,893.00 | 6.08 | 311.25 | 870.53 |
| EVM |  | 8,405 | 6,487.29 | 3,394.76 | 14.44 | 235.17 | 230.18 |
| Pasa-update* | | 8,299 | 6,427.86 | 1,728.09 | 7.34 | 235.43 | 741.27 |
| Final set** | | 8,104 | 6,516.11 | 1,743.16 | 7.43 | 234.69 | 742.59 |

Note: * contains the UTR area and the rest are not included. **This Final set contains the UTR region, which is obtained by removing the longest transcript from the variable cut by the PASA2 update result and filtering it to remove the redundant single exon. Filtration conditions: with TE greater than or equal to 20% overlap, early termination, only denovo evidence support and RPKM expression in less than 1 in 12 tissues. Annotated species include *Echinococcus multilocularis* (Emu), *Taenia solium* (Tso), *Hymenolepis microstoma* (Hmi), *Taenia asiatica* (Tas), *Taenia saginata* (Tsa), *Echinococcus granulosus* (Egr), *Macrostomumlignano* (Mli), *Schistosoma mansoni* (Sma), *Homo sapiens* (Hsa), *Canis lupus* (Clu), *Mus musculus* (Mmu) and *Ovisaries* (Oar) 12 species.

Supplementary Table 7. Alignment statistics with known protein libraries

|  | Number | Percent(%) |
| --- | --- | --- |
| Total | 8,104 | - |
| Swissprot | 6,287 | 77.60 |
| Nr | 7,908 | 97.60 |
| KEGG | 5,949 | 73.40 |
| InterPro | 6,514 | 80.40 |
| GO | 4,991 | 61.60 |
| Pfam | 6,057 | 74.70 |
| Annotated | 7,914 | 97.70 |
| Unannotated | 190 | 2.30 |

Supplementary Table 8. Non-coding RNA classification statistics

|  | Type | Copy (w*) | Average length (bp) | Total length (bp) | % of genome |
| --- | --- | --- | --- | --- | --- |
| miRNA | | 3 | 81.67 | 245 | 0.000172 |
| tRNA | | 126 | 75.74 | 9,543 | 0.006710 |
|  | rRNA | 7 | 84.14 | 589 | 0.000414 |
|  | 18S | 4 | 71.50 | 286 | 0.000201 |
| rRNA | 28S | 1 | 104 | 104 | 0.000073 |
|  | 5.8S | 1 | 106 | 106 | 0.000075 |
|  | 5S | 1 | 93 | 93 | 0.000065 |
|  | snRNA | 30 | 166.60 | 4,998 | 0.003514 |
| snRNA | CD-box | 2 | 92.50 | 185 | 0.000130 |
|  | HACA-box | 0 | 0 | 0 | 0 |
|  | splicing | 28 | 171.89 | 4,813 | 0.003384 |

Note: w* whole genome annotation, comprehensive data to calculate the average length and total length.

Supplementary Table 9**.** Genes used for gene family clustering in each species.

| **Species** | **Name** | **Gene** |
| --- | --- | --- |
| Mex | *Moniezia expansa* | 8,104 |
| Tsp | *Trichinella spiralis* | 14,616 |
| Cel | *Caenorhabditis elegans* | 20,060 |
| Sja | *Schistosoma japonicum* | 10,778 |
| Sme | *Schmidtea mediterranea* | 28,4070 |
| Sma | *Schistosoma mansoni* | 10,120 |
| Hna | *Hymenolepis nana* | 13,658 |
| Hta | *Hydatigera taeniaeformis* | 11,500 |
| Egr | *Echinococcus granulosus* | 10,251 |
| Emu | *Echinococcus multilocularis* | 10,618 |
| Tsa | *Taenia saginata* | 12,787 |
| Tas | *Taenia asiatica* | 13,222 |
| Hmi | *Hymenolepis microstoma* | 12,189 |

Supplementary Table 12**.** Agriculture and Rural Food Quality Supervision, Inspection and Testing Center (Shihezi)Test result report

№　W190153~154

| Project name | Unit | Results |  |
| --- | --- | --- | --- |
| Butyric acid (C4:0) | g/100g | — |  |
| Caproic acid (C6:0) | g/100g | — |  |
| Caprylic acid (C8:0) | g/100g | — |  |
| Caprylic acid (C10:0) | g/100g | — |  |
| Undecanoic acid (C11:0) | g/100g | — |  |
| Laurie acid (C12:0) | g/100g | — |  |
| Tridecanoic acid (C13:0) | g/100g | — |  |
| Myristic acid (C14:0) | g/100g | 0.00790 |  |
| Myristolcic acid (C14:1) | g/100g | — |  |
| Pentadecanoic acid (C15:0) | g/100g | 0.00206 |  |
| cis-10-Pentadecenoic acid (C15:1) | g/100g | — |  |
| Palmitic acid (Hexadacanoic acid) (C16:0) | g/100g | 0.0502 |  |
| Palmitoleic acid (C16:1) | g/100g | 0.00274 |  |
| Heptadecauoic acid (C17:0) | g/100g | 0.00234 |  |
| cis-10-Heptadecenoic acid (C17:1) | g/100g | 0.00150 |  |
| Stearic acid (Octadecanoic acid) (C18:0) | g/100g | 0.0456 |  |
| Elaidic acid (C18:1n9t) | g/100g | — |  |
| Oleic acid (Octadecenoic acid) (C18:1n9c) | g/100g | 0.0856 |  |
| Linolelaidic acid (C18:2n6t) | g/100g | 0.00592 |  |
| Linoleic acid（C18:2n6c） | g/100g | 0.120 | |
| γ-Linoleuic acid (C18:3n6) | g/100g | — | |
| a -Linolenic acid (C18:3n3) | g/100g | 0.0168 | |
| Arachidic acid (C20:0) | g/100g | 0.00614 | |
| cis-11-Eicosenoic acid（C20:1n9） | g/100g | — | |
| cis- 11 ;14-Eicosadienoic acid（C20:2） | g/100g | — | |
| cis-8, 11, 14-Eicosatrienoic acid（C20:3n6） | g/100g | — | |
| Henicosanoic acid（C21：0） | g/100g | — | |
| cis-11,14,17-Eicosatrienoic acid（C20:3n3） | g/100g | — | |
| Arachidonic acid（C20:4n6） | g/100g | 0.0548 | |
| cis-5,8, 11, 14, 17-Eicosapenraenoic acid (C20:5n3) | g/100g | — | |
| Behenic acid（C22:0） | g/100g | 0.00495 | |
| Erucic acid (C22:1n9) | g/100g | — | |
| cis- 13, 16-Docosadienoic acid（C22:2） | g/100g | — | |
| Tricosanoic acid（C23:0） | g/100g | — | |
| cis-4,7,10,13, 16, 19-Docosahexaenoic acid (C22:6n3) | g/100g | 0.00294 | |
| Lignoceric acid（C24:0） | g/100g | — | |
| Nervonic acid (C24:1n9) | g/100g | — | |

Supplementary Table 13**.** The functional references corresponding to the homeobox genes

| Genes | References |
| --- | --- |
| *CDX* | Beck F, & Stringer EJ. The role of Cdx genes in the gut and in axial development. Biochem Soc T 2010;38:353-357. |
| *Pou5* | Kotkamp K, Kur E, Wendik B, et al. Pou5f1/Oct4 Promotes Cell Survival via Direct Activation of mych Expression during Zebrafish Gastrulation. PLoS ONE 2014;9: e92356. |
| *Hox5* | Hrycaj SM, Dye BR, Baker NC, L et al. Hox5 Genes Regulate the Wnt2/2b-Bmp4-Signaling Axis during Lung Development. Cell Rep 2015;12:903-912. |
| *Pitx* | Chen Y, Knösel T,Ye F, et al. Decreased PITX1 homeobox gene expression in human lung cancer. Lung Cancer 2007;55:287-94. |
| *Hox9-13* | Drake KA, Adam M, Mahoney R, et al. Disruption of Hox9,10,11 function results in cellular level lineage infidelity in the kidney. Sci Rep 2018;8:6306. |
| *Pax2\8* | Grote D, Souabni A, Busslinger M, et al. Pax2/8-regulated Gata3 expression is necessary for morphogenesis and guidance of the nephric duct in the developing kidney. Development 2005;133:53-61. |
| *Hnf1* | Lokmane L, Heliot C, Garcia-Villalba P, et al. vHNF1 functions in distinct regulatory circuits to control ureteric bud branching and early nephrogenesis. Development 2010;137:347-357. |
| *Pdx* | Ashizawa SMD, Brunicardi FC, Wang XP. PDX-1 and the Pancreas. Pancreas 2004; 288:109-120. |
| *Hhex* | Gauvrit S, Villasenor A, Strilic B, et al. HHEX is a transcriptional regulator of the VEGFC/FLT4/PROX1 signaling axis during vascular development. Nat Commun 2018;9:2704. |
| *Isl* | Zhang Q, Yang Z, Wang W, et al. A positive feedback regulation of ISL-1 in DLBCL but not in pancreatic β-cells. *Biochem Biophys Res Commun*. 2014;449:295-300. |
| *Hox3* | Ma LH, Grove CL, Baker R. Development of oculomotor circuitry independent of hox3 genes. *Nat Commun*. 2014;5:4221. |
| *Six1/2* | Vocking O, Kourtesis I, Hausen H. Posterior eyespots in larval chitons have a molecular identity similar to anterior cerebral eyes in other bilaterians. Evol Dev 2015;6:40. |
| *Pax6* | Grindley JC，Davidson DR，Hill RE．The role of Pax6 in eye and nasal development．Development 1995;121:1433-1442 |
| *Vsx* | Erclik T, Hartenstein V, Lipshitz HD, et al. Conserved role of the Vsx genes supports a monophyletic origin for bilaterian visual systems. *Curr Biol*. 2008;18:1278-1287. |
| *Emx* | Sen S, Reichert H, VijayRaghavan K. Conserved roles of ems/Emx and otd/Otx genes in olfactory and visual system development in Drosophila and mouse. *Open Biol*. 2013;3:120177. |
| *Prop* | Ahijevych K, Tepper BJ, Graham MC, et al. Relationships of PROP Taste Phenotype, Taste Receptor Genotype, and Oral Nicotine Replacement Use. Nicotine Tob Res 2015;17:1149-1155. |
| *Prox* | Jeffery W, Strickler A, Guiney S, et al. Prox 1 in eye degeneration and sensory organ compensation during development and evolution of the cavefish Astyanax. *Dev Genes Evol*. 2000;210:223-230. |
| *Lhx6* | Cesario JM, Landin Malt A, Deacon LJ, et al. Lhx6 and Lhx8 promote palate development through negative regulation of a cell cycle inhibitor gene, p57Kip2. *Hum Mol Genet*. 2015;24:5024-5039. |
| *Mkx* | Kayama T, Mori M, Ito Y, et al. Gtf2ird1-Dependent Mohawk Expression Regulates Mechanosensing Properties of the Tendon. *Mol Cell Biol*. 2016;36:1297-1309. |
| *Tshz* | Coré N, Caubit X, Metchat A, et al. Tshz1 is required for axial skeleton, soft palate and middle ear development in mice. *Dev Biol*. 2007;308:407-420. |
| *Pax3\7* | Camporez JP, Petersen MC, Abudukadier A, et al. Anti-myostatin antibody increases muscle mass and strength and improves insulin sensitivity in old mice. *Proc Natl Acad Sci U S A*. 2016;113:2212-2217. |
| *Otp* | Wircer E, Blechman J, Borodovsky N, et al. Homeodomain protein Otp affects developmental neuropeptide switching in oxytocin neurons associated with a long-term effect on social behavior. *Elife*. 2017;6:e22170. |
| *Pax4* | Lin HT, Kao CL, Lee KH, et al. Enhancement of insulin-producing cell differentiation from embryonic stem cells using pax4-nucleofection method. *World J Gastroenterol*. 2007;13:1672-1679. |
| *Pou1* | Rosenfeld MG. ROU-domain transcription factors: power-ful developmental regulators. Gens Development,1991,5: 897- 907. |
| *Six4/5* | Schlosser G. Induction and specification of cranial placodes. Dev Biol 2006;294:303-51. |
| *Lhx2\9* | Chou SJ, Perez-Garcia CG, Kroll TT, et al. Lhx2 specifies regional fate in Emx1 lineage of telencephalic progenitors generating cerebral cortex. *Nat Neurosci*. 2009;12:1381-1389. |
| *Shox* | OgataT, OnigataK, HotsuboT, et al. Growth hormone and gonado-tropin-releasing hormone analog the rapyinhaploin sufficiency of SHOX. Endocr J 2009;48:317-322. |
| *Uncx* | Daniele G, Simonetti G, Fusilli C, et al. Epigenetically induced ectopic expression of UNCX impairs the proliferation and differentiation of myeloid cells. *Haematologica*. 2017;102:1204-1214. |
| *Zeb* | Shin JO, Kim EJ, Cho KW, et al. BMP4 signaling mediates Zeb family in developing mouse tooth. *Histochem Cell Biol*. 2012;137:791-800. |
| *Otx* | Nishida A，Furukawa A，Koike C，et al．Otx2 homeobox gene controls retinal photoreceptor cell fate and pineal gland development． Nat Neurosci 20036:1255-1263． |
| *Evx* | Kozin VV, Filimonova DA, Kupriashova EE, et al．Mesoderm patterning and morphogenesis in the polychaete Alitta virens (Spiralia, Annelida): Expression of mesodermal markers Twist, Mox, Evx and functional role for MAP kinase signaling. *Mech Dev*. 2016;140:1-11. |
| *Dlx* | Fazel Darbandi S, Poitras L, Monis S, et al. Functional consequences of I56ii Dlx enhancer deletion in the developing mouse forebrain. *Dev Biol*. 2016;S0012-1606(16)30263-9. |
| *Uncx* | Sammeta N, Hardin DL, McClintock TS. Uncx regulates proliferation of neural progenitor cells and neuronal survival in the olfactory epithelium. *Mol Cell Neurosci*. 2010;45:398-407. |
| *Lbx* | Chen XW, Lou QY, He JY, et al. Role of Zebrafish Lbx2 in Embryonic Lateral Line Development. PLoS ONE 2011;6:e29515. |
| *Prrx* | Takacs CM, Moy VN, Peterson KJ. Testing putative hemichordate homologues of the chordate dorsal nervous system and endostyle: expression of *NK2.1 (TTF-1)* in the acorn worm *Ptychodera flava* (Hemichordata, Ptychoderidae). Evol Dev 2002;4:6405-417. |
| *Pou4* | Zhang L, Wahlin K, Li YY, et al. RIT2, a neuron-specific small guanosine triphosphatase, is expressed in retinal neuronal cells and its promoter is modulated by the POU4 transcription factors. Molecular Vision 2013;19:1371-1386. |
| *Pou6* | Wollesen T, McDougall C, Degnan BM, et al. POU genes are expressed during the formation of individual ganglia of the cephalopod central nervous system. EvoDevo 2014;5:41. |
| *Rax* | Irie S, Sanuki R, Muranishi Y, et al. Rax Homeoprotein Regulates Photoreceptor Cell Maturation and Survival in Association with Crx in the Postnatal Mouse Retina. Mol Cell Biol 2015;35:2583-2596. |
| *Gsx* | Chapman H, Riesenberg A, Ehrman LA, et al. Gsx transcription factors control neuronal versus glial specification in ventricular zone progenitors of the mouse lateral ganglionic eminence. Dev Biol 2018;442:115-126. |
| *Meox* | Dong K, Guo X, Chen W, et al. Mesenchyme homeobox 1 mediates transforming growth factor-beta (TGF-beta)-induced smooth muscle cell differentiation from mouse mesenchymal progenitors. J Biol Chem 2018;293:8712-8719. |
| *Gbx* | Wollesen T, Scherholz M, Rodriguez Monje SV, et al. Brain regionalization genes are co-opted into shell field patterning in Mollusca. Sci Rep 2017;7:5486. |
| *ISX* | Wang LT, Chiou SS, Chai CY, et al. Intestine-Specific Homeobox Gene *ISX* Integrates IL6 Signaling, Tryptophan Catabolism, and Immune Suppression. *Cancer Res*. 2017;77:4065-4077. |
| *Rhox* | Borgmann J, Tüttelmann F, Dworniczak B, et al. The human RHOX gene cluster: target genes and functional analysis of gene variants in infertile men. *Hum Mol Genet*. 2016;25:4898-4910. |
| *Barx2* | Makarenkova HP, Meech R. Barx homeobox family in muscle development and regeneration. *Int Rev Cell Mol Biol*. 2012;297:117-173. |

Supplementary Table 14. The specific conserved domains/motifs numbers of germ cell marker family

| **Gene Family** | **Subfamily** | **Conserved domain/motif** |
| --- | --- | --- |
| Dicer | - | PF03368.14:Dicer_dimer |
| Drosha | - | PF00035.26:dsrm |
| Nanos | - | PF05741.13:zf-nanos |
| Pumilio | - | PF00806.19:PUF |
| AGO | - | PF08699.10:ArgoL1 PF02170.22:PAZ PF16488.5:ArgoL2 PF16487.5:ArgoMid PF02171.17:Piwi |
| Piwi/Piwi-like | - | PF02171.17:Piwi |
| Royal | PWWP | PF00855.17:PWWP |
|  | Agenet | PF05641.12:Agenet |
|  | CHROMO | PF00385.24:Chromo |
|  | MBT | PF02820.18:MBT |
|  | Tudor | PF00567.24:TUDOR |
| DEAD-Box Helicase | Vasa | DEAD  AXTGXGKT  SAT  PTREL  TPGR  GG  A/SRGXD  HRIGR/YXHRXGRXXR |
|  | PL10 |  |
|  | DDX5 |  |

Supplementary Table 15. Components of *M. expansa* Wnt, Hedgehog and Notch signalling pathways

| Gene_ID | Description |
| --- | --- |
| Wnt |  |
| evm.model.Contig71.1043 | Wnt-11 |
| evm.model.Contig512.31 | Wnt-4 |
| evm.model.Contig51.111 | Wnt-11b |
| evm.model.Contig60.191 | Wnt-1 |
| evm.model.Contig75.151 | Wnt-2b-A |
| evm.model.Contig75.186 | Wnt-5b |
| evm.model.Contig82.193 | Frizzled-4 |
| evm.model.Contig82.627 | Transcription factor 7 |
| evm.model.Contig51.380 | Transforming protein RhoA |
| evm.model.Contig52.161 | Transcription factor 7-like 2 |
| evm.model.Contig96.252 | Transcription factor jun-1 |
| evm.model.Contig63.78 | Transcription factor jun-B |
| evm.model.Contig63.79 | Transcription factor jun-B |
| evm.model.Contig82.66 | G1/S-specific cyclin-D1 |
| evm.model.Contig60.151 | gsk3b; glycogen synthase kinase-3 beta |
| evm.model.Contig52.612 | CTNNB1 binding, N-teminal |
| evm.model.Contig82.318 | Segment polarity protein dishevelled homolog DVL-3 |
| evm.model.Contig82.318 | Segment polarity protein dishevelled homolog |
| evm.model.Contig1.103 | Ras-related C3 botulinum toxin substrate 1 |
| evm.model.Contig60.42 | Ras-related C3 botulinum toxin substrate 2 |
| evm.model.Contig52.499 | jnk:sapk associated protein |
| evm.model.Contig91.40.1 | JNK interacting protein |
| evm.model.Contig75.448.1 | JNK/Rab-associated protein-1, N-terminal |
| evm.model.Contig51.303 | JNK/Rab-associated protein-1, N-terminal |
| evm.model.Contig75.187 | 1-phosphatidylinositol 4,5-bisphosphate phosphodiesterase beta-4 |
| evm.model.Contig82.47 | Calcineurin subunit B type 1 |
| evm.model.Contig63.290 | Serine/threonine-protein phosphatase 2B catalytic subunit alpha isoform |
| evm.model.Contig52.629 | Serine/threonine-protein phosphatase 2B catalytic subunit alpha isoform |
| evm.model.Contig61.46 | PRKCA-binding protein |
| evm.model.Contig68.28 | Protein kinase C beta type |
| TGF-β |  |
| evm.model.Contig493.87 | Bone morphogenetic protein 7 |
| evm.model.Contig96.235 | Growth/differentiation factor 5 |
| evm.model.Contig71.1049 | Activin receptor type-1 |
| evm.model.Contig75.211 | Activin receptor type-1 |
| evm.model.Contig71.202 | Activin receptor type-2A |
| evm.model.Contig69.125 | Mothers against decapentaplegic homolog 2 |
| evm.model.Contig69.554 | Mothers against decapentaplegic homolog 3 |
| evm.model.Contig512.205 | Mothers against decapentaplegic homolog 4 |
| evm.model.Contig71.911 | Mothers against decapentaplegic homolog 9 |
| evm.model.Contig64.28 | ER membrane protein complex subunit 8 |
| evm.model.Contig82.537 | ER membrane protein complex subunit 7 |
| evm.model.Contig62.130 | ER membrane protein complex subunit 1 |
| evm.model.Contig63.35 | ER membrane protein complex subunit 4 |
| evm.model.Contig69.702 | ER membrane protein complex subunit 3 |
| evm.model.Contig69.584 | ER membrane protein complex subunit 10 |
| evm.model.Contig52.510 | ER membrane protein complex subunit 2 |
| evm.model.Contig52.455 | Inhibin beta A chain |
| evm.model.Contig68.47 | Nodal modulator 2 |
| evm.model.Contig52.293 | Retinoblastoma-like protein 1 |
| evm.model.Contig82.237 | Transcription factor Dp-1 |
| evm.model.Contig61.86 | Transcription factor E2F4 |
| evm.model.Contig71.150 | Transforming growth factor-beta-induced protein ig-h3 |
| evm.model.Contig51.402 | Transforming growth factor beta-1-induced transcript 1 protein |
| evm.model.Contig69.581 | Transforming growth factor-beta receptor-associated protein 1 |
| evm.model.Contig69.582 | Transforming growth factor-beta receptor-associated protein 1 |
| evm.model.Contig69.49 | Transforming growth factor-beta-induced protein ig-h3 |
| evm.model.Contig79.145 | Transforming growth factor beta-1-induced transcript 1 protein O |
| evm.model.Contig71.315 | TGF-beta receptor type-1 |
| evm.model.Contig71.316 | TGF-beta receptor type-1 |
| Hippo |  |
| evm.model.Contig70.114 | Baculoviral IAP repeat-containing protein 5.2 |
| evm.model.Contig82.66 | G1/S-specific cyclin-D1 |
| evm.model.Contig512.196 | Connective tissue growth factor |
| evm.model.Contig75.109 | FERM domain-containing protein 6 |
| evm.model.Contig4.66 | MOB kinase activator 1A |
| evm.model.Contig512.205 | Mothers against decapentaplegic homolog 4 |
| evm.model.Contig1.52 | Zinc finger protein SNAI2 |
| evm.model.Contig71.586 | Transcription factor SOX-2 |
| evm.model.Contig82.223 | Merlin |
| evm.model.Contig71.1412 | Serine/threonine-protein phosphatase 2A catalytic subunit alpha isoform |
| evm.model.Contig78.72 | Serine/threonine-protein phosphatase 2A 65 kDa regulatory subunit A beta isoform |
| evm.model.Contig63.303 | Serine/threonine-protein phosphatase 2A 65 kDa regulatory subunit A alpha isoform |
| evm.model.Contig60.7 | Serine/threonine-protein phosphatase 2A 65 kDa regulatory subunit A alpha isoform |
| evm.model.Contig69.349 | Serine/threonine-protein phosphatase 2A 55 kDa regulatory subunit B alpha isoform |
| evm.model.Contig70.146 | Serine/threonine-protein phosphatase 2A 55 kDa regulatory subunit B alpha isoform |
| evm.model.Contig82.591 | Serine/threonine-protein kinase 3 |
| evm.model.Contig70.35 | Serine/threonine-protein kinase 36 |
| evm.model.Contig71.939 | Serine/threonine-protein kinase 38-like |
| evm.model.Contig96.240 | Transcriptional enhancer factor TEF-3 |
| evm.model.Contig63.237 | Transcriptional enhancer factor TEF-3 |
| evm.model.Contig75.352 | transcriptional coactivator YAP1 |
| Hedgehog |  |
| evm.model.Contig512.244.1 | Beta-arrestin-1 |
| evm.model.Contig71.770 | Beta-arrestin-1 |
| evm.model.Contig71.771 | Beta-arrestin-1 |
| evm.model.Contig60.241.9 | Apoptosis regulator Bcl-2 |
| evm.model.Contig82.66 | G1/S-specific cyclin-D1 |
| evm.model.Contig82.547 | BTB/POZ domain-containing adapter for CUL3-mediated RhoA degradation protein 3 |
| evm.model.Contig63.259 | Cullin-3 |
| evm.model.Contig82.141 | Protein patched homolog 1 |
| evm.model.Contig71.634 | Suppressor of fused homolog |


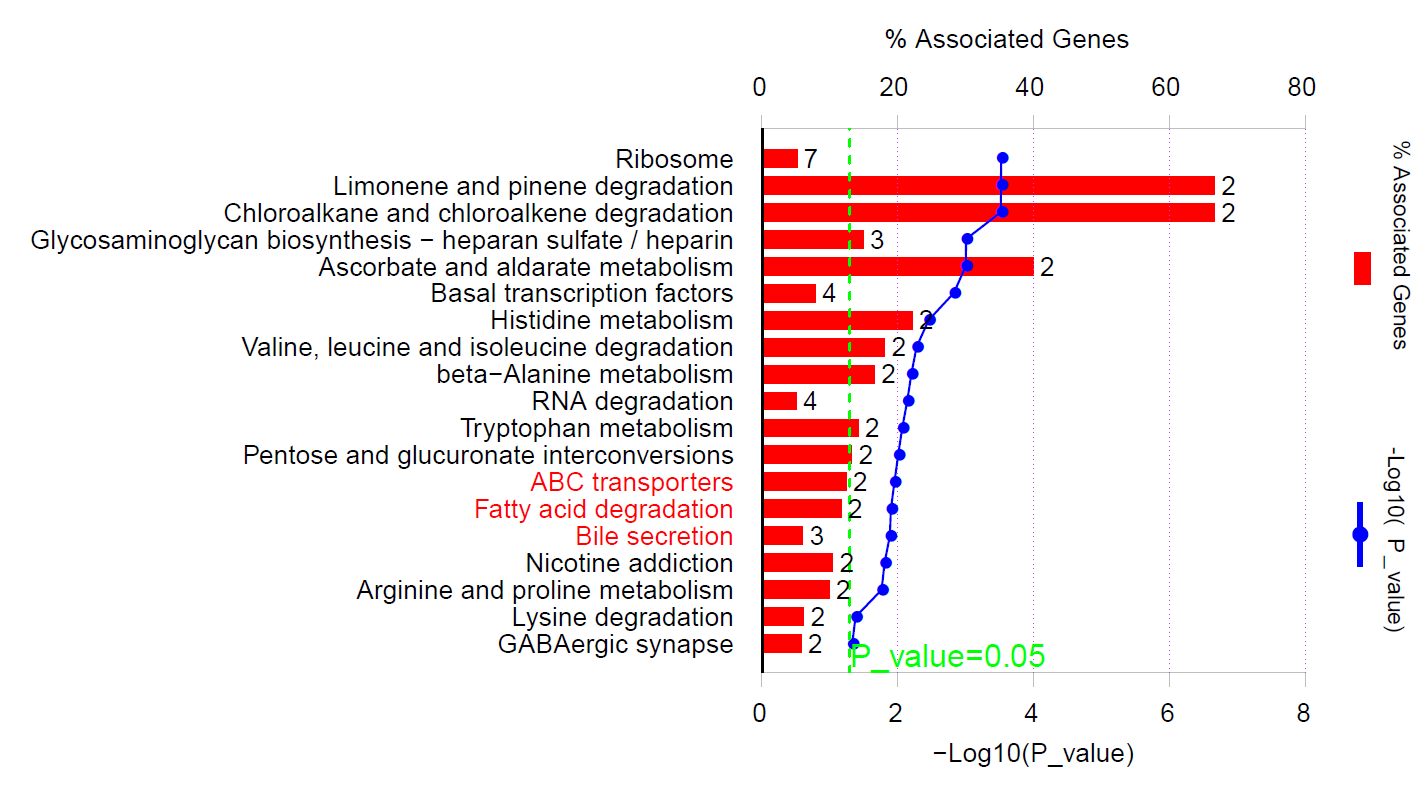


Supplementary Fig 1**.** KEGG enrichment results of *M. expansa* unique genes. The upper coordinate refers to the ratio of the number of enriched genes to the entire pathway genes; the lower coordinate refers to -log10 (P_value); the blue broken line is -log10 (P_value); the red column refers to the upper coordinate refers to the ratio of the number of enriched genes to the entire pathway genes, and the number refers to the number of enriched genes.


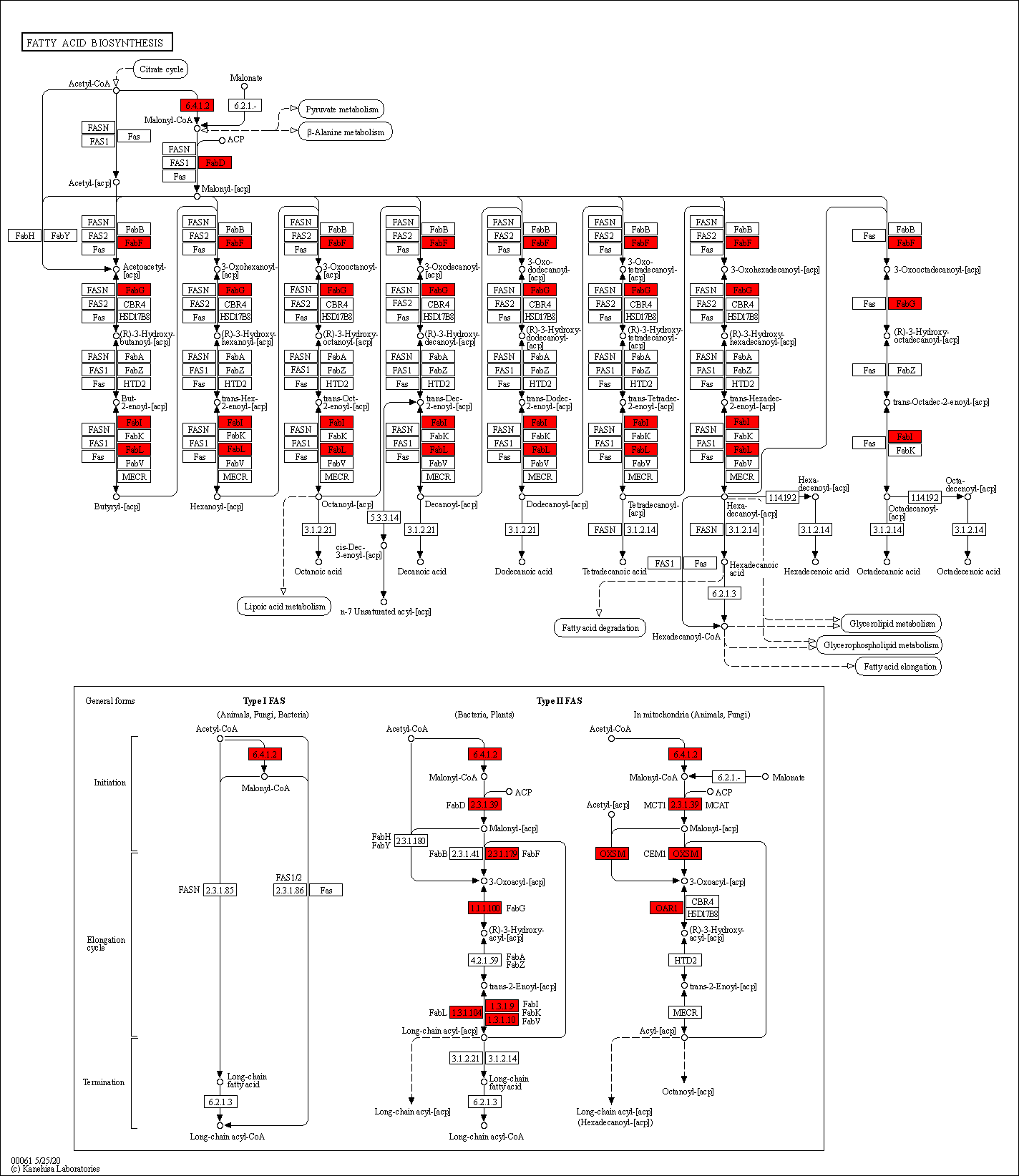


Supplementary Fig 2**.** Fatty acid synthesis in the *M. expansa*. Red is the gene in *M. expansia* and white is the reference KEGG pathway downloaded from KEGG (https://www.kegg.jp/).


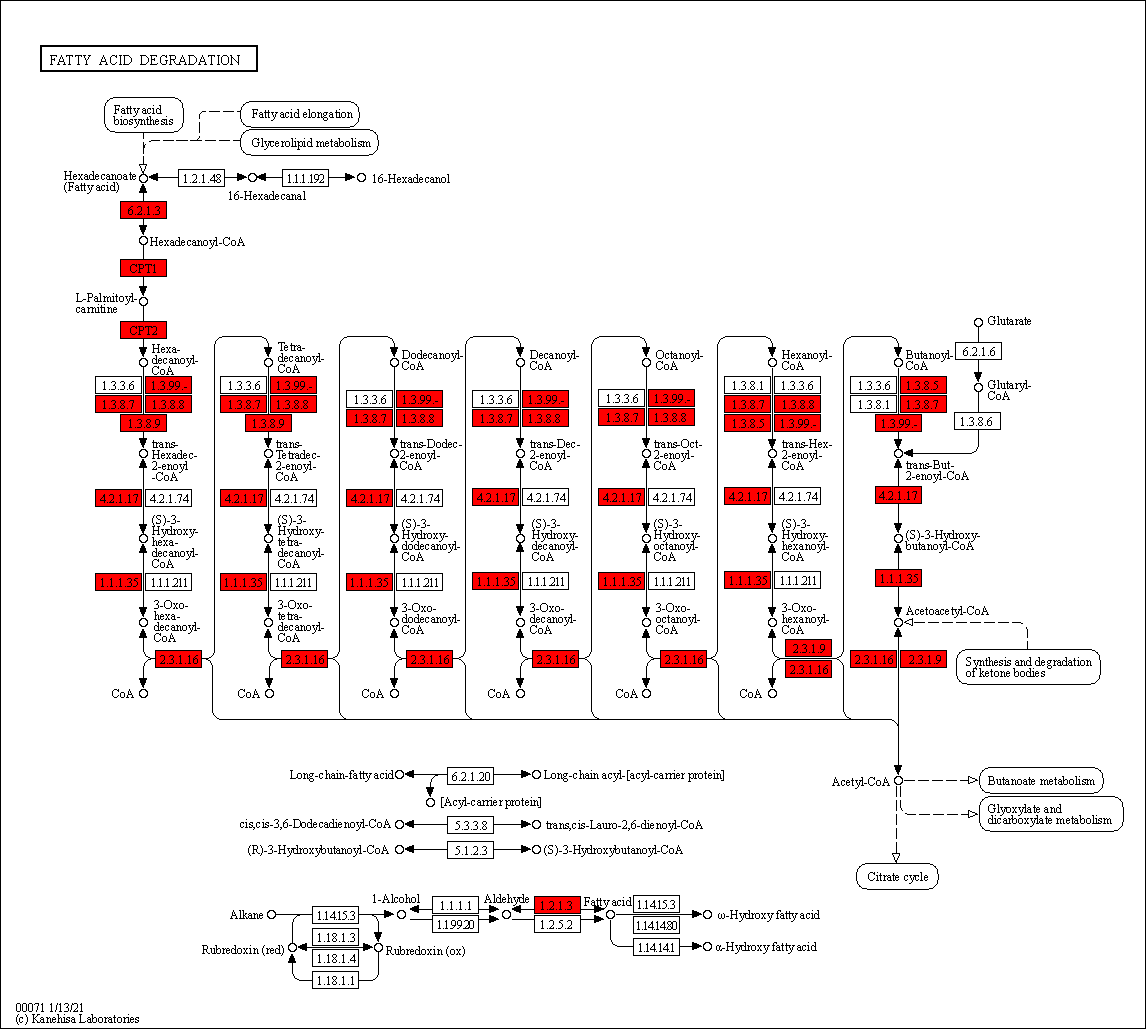


Supplementary Fig 3**.** Fatty acid degradation in the *M. expansa*.


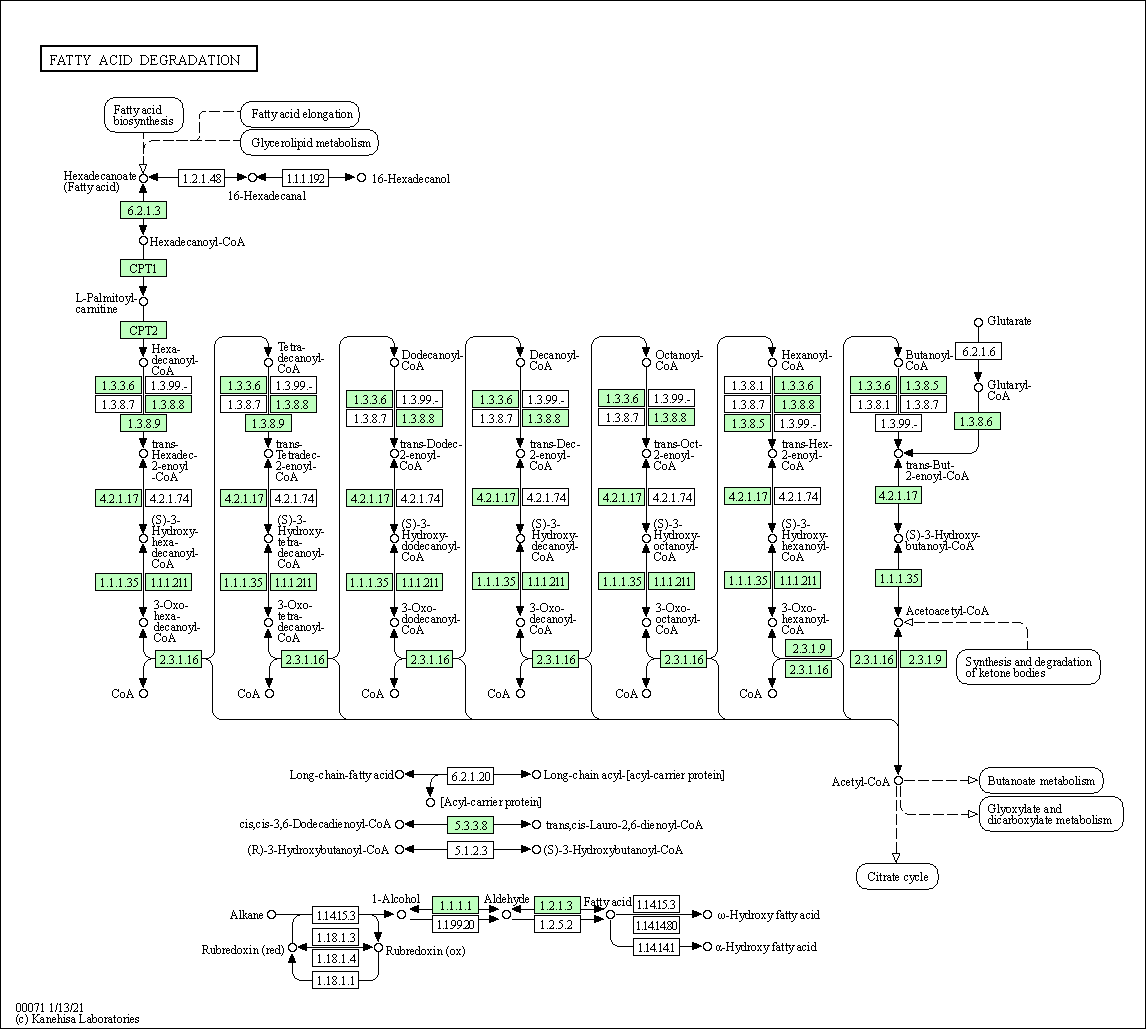


Supplementary Fig 4**.** Fatty acid degradation in the *O. viverrini*.


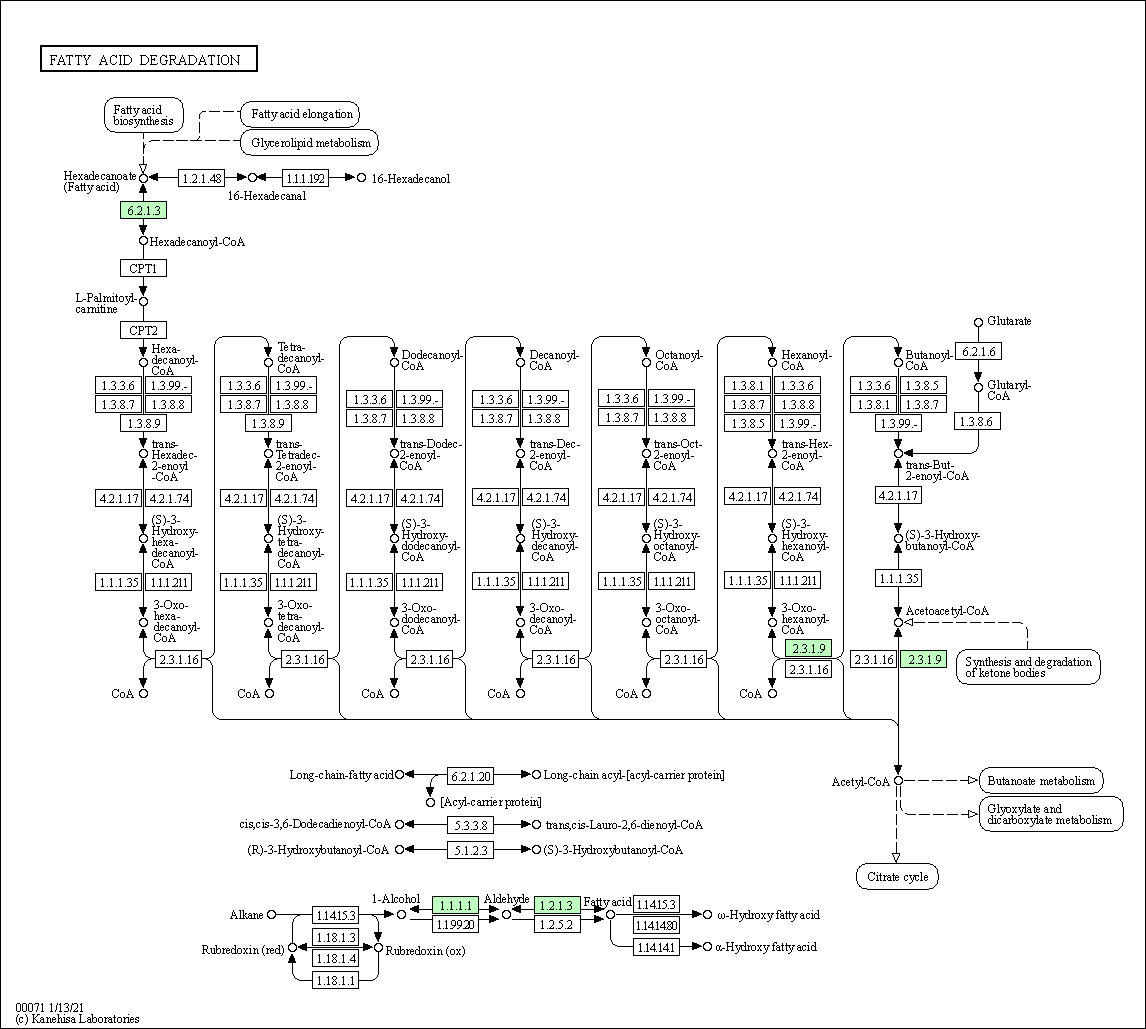


Supplementary Fig 5**.** Fatty acid degradation in the *Schistosoma mansoni*.


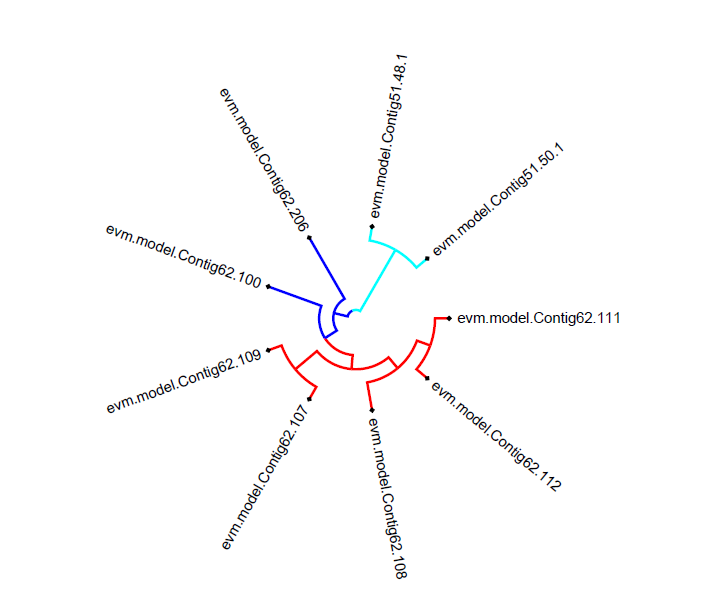


Supplementary Fig 6**.** FABP Subfamily classification in *M. expansa***.** Dark blue represents subfamily II, red represents subfamily III, light blue represents subfamily IV.


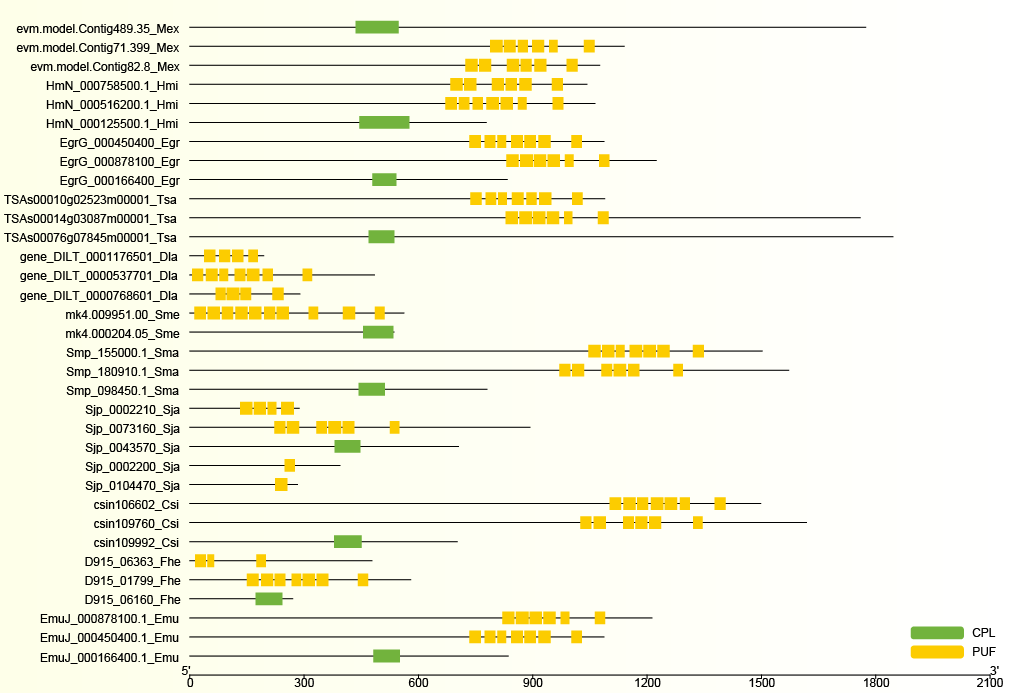


Supplementary Fig 7**.** PUF structure statistics of Pumillo gene family


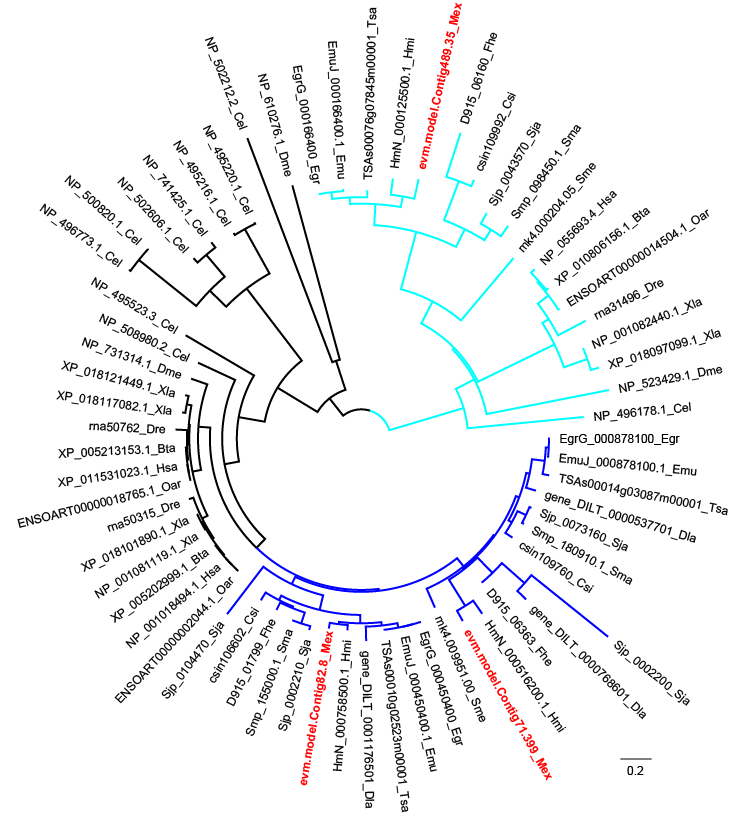


Supplementary Fig 8**.** Phylogenetic tree of pumillo family of various species. Dark blue represents platyhelminths species branch, black represents species that do not contain platyhelminths, light blue represents all species branch.


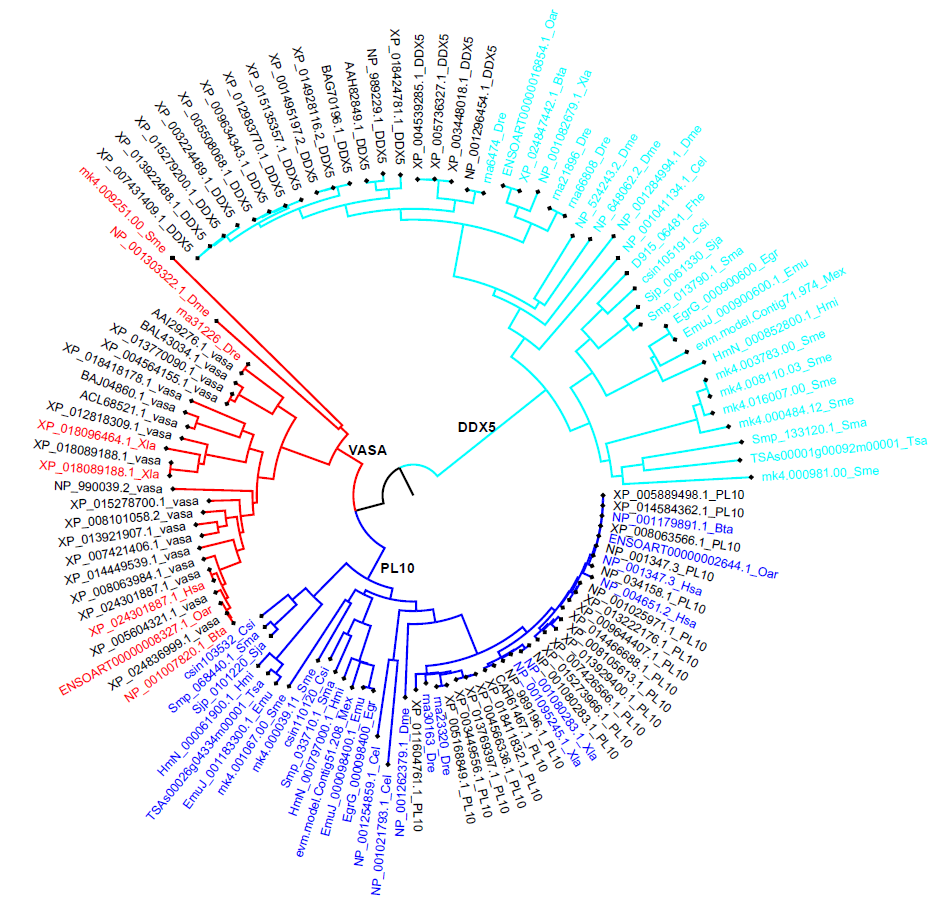


Supplementary Fig 9**.** Phylogenetic tree of DEAD_Box family of various species.


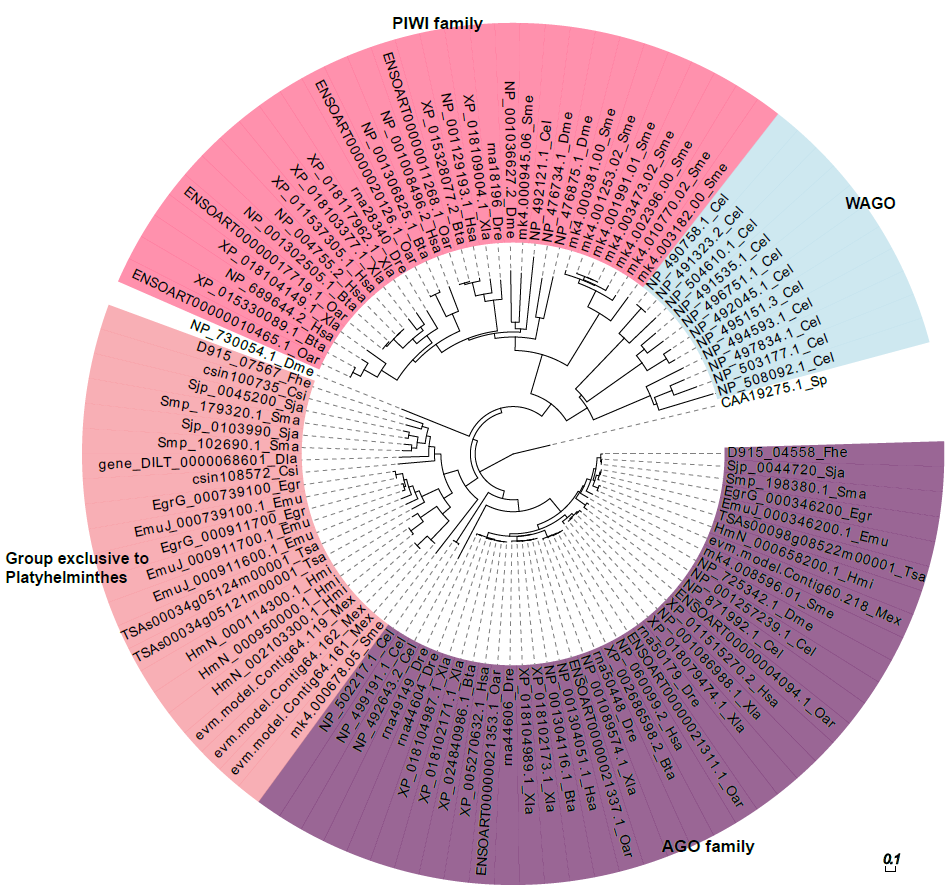


Supplementary Fig 10**.** Phylogenetic tree of Argonaute family of various species.


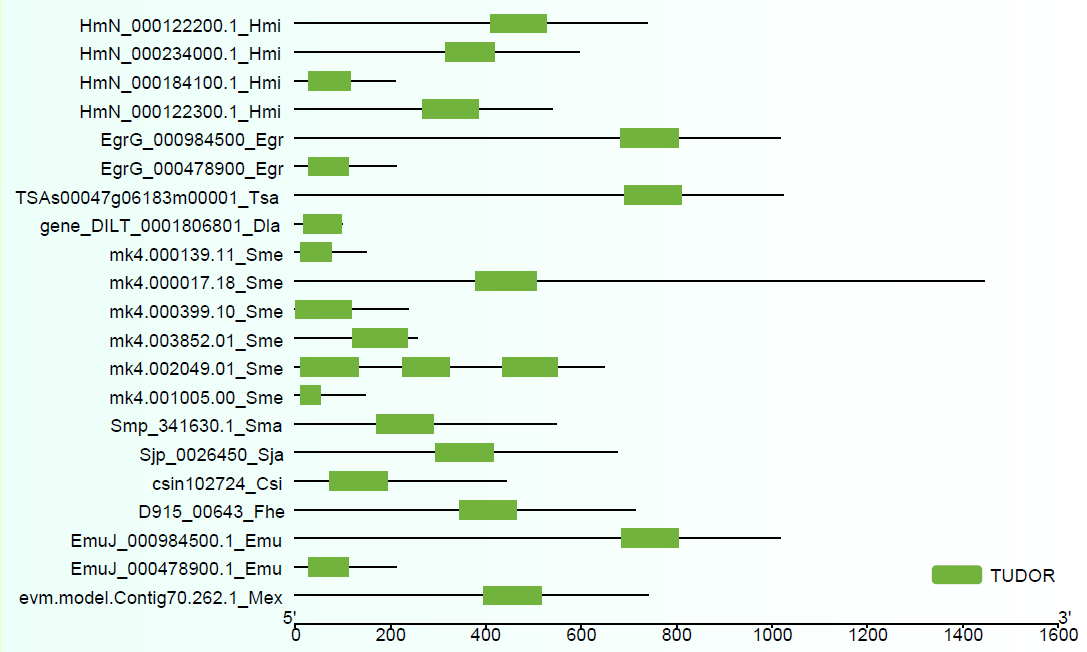


Supplementary Fig 11**.** Tudor family domain analysis


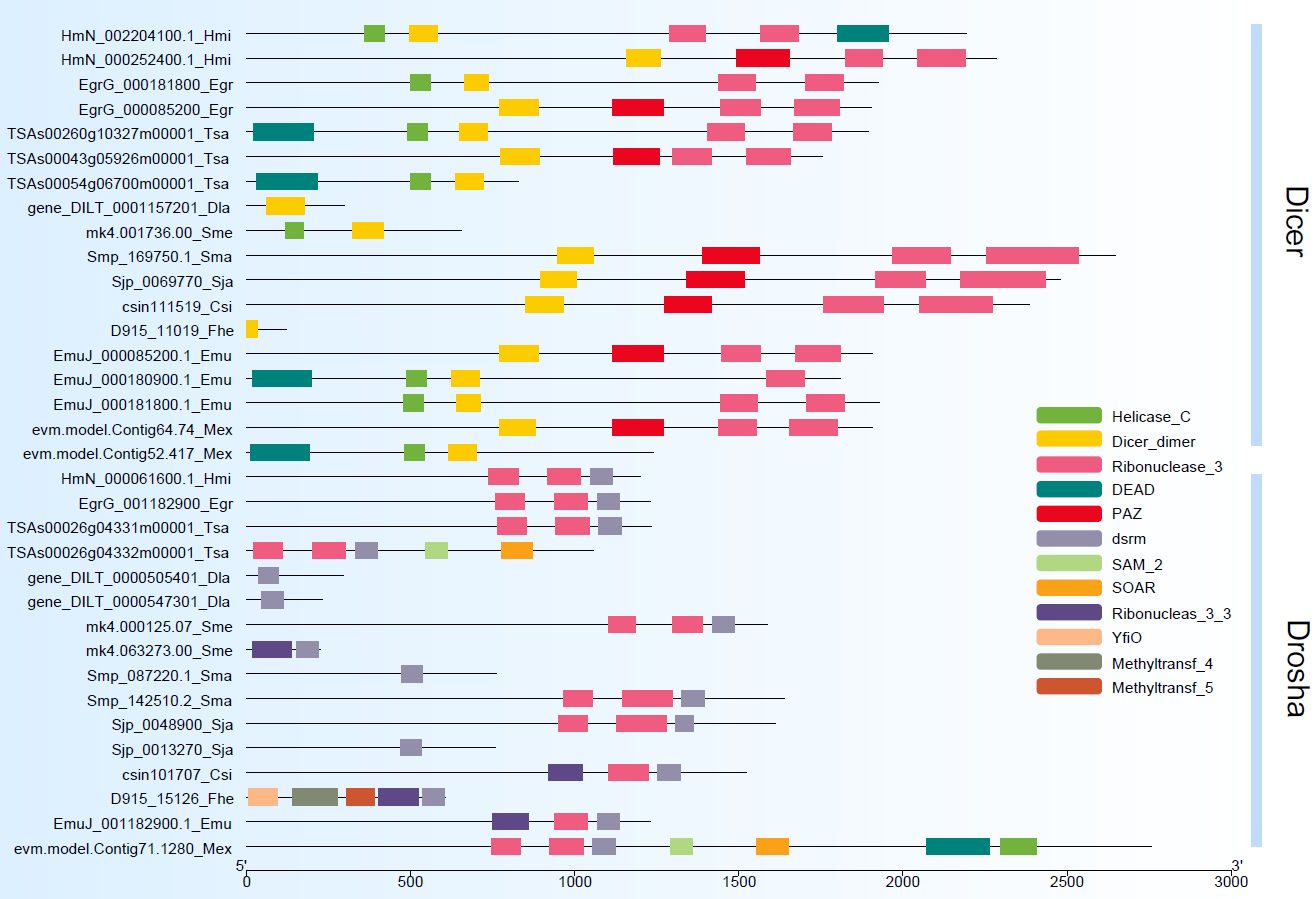
 Supplementary Fig 12**.** Dicer\Drosha family domain analysis
